# Aflatoxin M1 in Milk from South–Central and Northwest China: Prevalence and Integrated Risk Characterization for Different Age-Sex Groups of Consumers

**DOI:** 10.3390/foods15122102

**Published:** 2026-06-11

**Authors:** Xue Zhang, Ting Zhang, Jie Zhang, Yinsheng Qiu, Qirong Lu, Jianglin Xiong, Chong Wang

**Affiliations:** 1 School of Animal Science and Nutrition Engineering, Wuhan Polytechnic University, Wuhan 430023, China; 2 Key Laboratory of Applied Technology on Green-Eco-Healthy Animal Husbandry of Zhejiang Province, College of Animal Science and Technology & College of Veterinary Medicine, Zhejiang A&F University, Hangzhou 311300, China

**Keywords:** aflatoxin M1, milk, prevalence, risk characterization, China

## Abstract

Aflatoxin M1 (AFM1) is a Group 1 human carcinogen that poses a public health risk through milk contamination. This study investigated AFM1 contamination in pasteurized and ultra-high temperature (UHT) milk from south–central and northwest China, and assessed the associated health risks for consumers based on age, sex, and milk consumption scenarios. In total, 902 milk samples (493 pasteurized, 409 UHT) were collected during the summer and winter seasons of 2023–2024. AFM1 levels were determined using a validated enzyme-linked immunosorbent assay. AFM1 was detected in 75.39% of samples (mean concentration, 12.35 ± 10.27 ng/L; maximum, 75.57 ng/L). While 1.55% of samples exceeded the EU maximum limit (50 ng/L), all complied with the Chinese limit (500 ng/L). Contamination was significantly higher in south–central China than northwest China, higher in winter than summer, and higher in pasteurized milk than UHT milk (*p* < 0.05). Preschool children in south–central China consuming 400 mL/day of milk are the group with the highest AFM11 exposure risk. And the margin of exposure and population risk of liver cancer suggested little carcinogenic risk. Therefore, targeted monitoring strategies for AFM1 in milk are recommended, along with dietary guidance for high-risk groups, especially vulnerable young children, to mitigate exposure.

## 1. Introduction

Aflatoxin M1 (AFM1) is a hydroxylated metabolite of the carcinogenic compound aflatoxin B1 (AFB1) produced mainly by two *Aspergillus* species (*A. flavus* and *A. parasiticus*) and has been established as a contaminate of milk and dairy products produced from dairy cattle consuming AFB1-contaminated feed [1]. Classified as a Group 1 human carcinogen [2], AFM1 poses a significant public health risk, which is particularly severe in populations with a high prevalence of hepatitis B virus (HBV) infection due to synergistic effects [3]. Chronic exposure to AFM1—even at low levels—is concerning, especially for children given their higher milk consumption relative to body weight [4]. To mitigate AFM1 exposure through milk consumption, regulatory limits have been established worldwide. For example, the European Union set a strict maximum level (ML) of AFM1 at 50 ng/L [5], whereas both China [6] and the United States [7] have set MLs at 500 ng/L.

Milk production in China increased from 36.827 million metric tons in 2021 to 39.316 in 2022, 41.976 in 2023, and 40.794 in 2024, demonstrating the heightened consumption of diverse dairy products—especially pasteurized and ultra-high-temperature (UHT) milk [8]. In addition, the Chinese government implemented a three-child policy and provided childcare subsidies in 2021, while the Chinese Society of Nutrition recommended that residents increase milk consumption in 2022 [9]. These new policies have greatly promoted the production and consumption of milk. Meanwhile, milk safety, particularly AFM1 contamination, has attracted significant attention from both the government and consumers. Given that China is a vast territory, AFM1 contamination of milk widely varies across different regions, as detection rates and levels are influenced by climatic conditions, seasons, feeding practices, and storage methods [10,11,12,13]. As important regions of milk production and consumption, northwest China is characterized by a dry temperate continental climate, whereas south–central China experiences a subtropical humid monsoon climate [13,14]. However, despite their distinct climatic conditions, direct comparisons of AFM1 contamination risks in dairy products between these two regions have rarely been conducted. Moreover, previous risk assessments of AFM1 in milk were based on a fixed consumption level [1,15,16], whereas risk assessment across varying intake amounts can better reveal dynamic changes. Thus, in-depth investigations and risk assessments of milk consumer intake are warranted to reveal contamination characteristics, guide safe dairy production, and mitigate AFM1 exposure.

Therefore, this study aims to (1) investigate variations in AFM1 levels in milk with respect to two regions (northwest and south–central China), two seasons (summer and winter), and two major milk types (pasteurized and UHT); (2) characterize the risk of AFM1 exposure with the margin of exposure (MOE) and population risk of liver cancer (PRLC) in 10 groups based on age and sex under different milk intake levels.

## 2. Materials and Methods

### 2.1. Milk Sample Collection

In total, 902 milk samples (493 pasteurized and 409 UHT) were collected from convenience stores, supermarkets, and milk stations in northwest China (Gansu province and Ningxia Hui autonomous region) and south–central China (Hubei and Hunan provinces) in 2023 and 2024. Among the 409 milk samples collected in northwest China, 208 were obtained in the summer (133 pasteurized and 75 UHT) and 201 in the winter (120 pasteurized and 81 UHT). Of the 493 milk samples collected in south–central China, 259 were obtained in the summer (133 pasteurized and 126 UHT) and 234 in the winter (107 pasteurized and 127 UHT). According to the information indicated on the packaging, the milk samples were produced on scheduled different dates. Each milk sample was thoroughly mixed, and 50 mL aliquots were dispensed into centrifuge tubes containing a preservative (Broad Spectrum Microtabs II; Advanced Instruments LLC, Norwood, MA, USA). After homogenization, the samples were labeled and stored at −20 °C until analysis for AFM1 content within the validity period.

### 2.2. Determination of AFM1 Content in Milk

AFM1 in milk samples was detected using an enzyme-linked immunosorbent assay (ELISA) (RIDASCREEN^®^ AFM1 ELISA test kit; catalog no. R1121; R-Biopharm AG, Darmstadt, Germany) in accordance with the manufacturer’s specifications. The ELISA kit features a limit of detection (LOD) of 5 ng/L for AFM1, 100% specificity for AFM1, and less than 10% cross-reactivity with AFM2. The kit includes six standard solutions (0, 5, 10, 20, 40, and 80 ng/L), which were used to establish a calibration curve for the quantification of AFM1 in milk samples in each analytical run. The assay procedure consisted of three major stages: sample pretreatment, detection of treated samples, and concentration calculation, as described in a previous report by our group [15]. Briefly, milk samples were thawed at room temperature (20–25 °C). Subsequently, a 7 mL aliquot of each sample was centrifuged at 3500× *g* for 10 min at 4 °C to obtain defatted milk. For the ELISA, pretreated samples and standards were added to antibody-coated wells and then incubated with the enzyme conjugate. After washing, substrate/chromogen solution was added and the reaction was stopped before measuring the absorbance at 450 nm using a microplate reader (SpectraMax i3x; Molecular Devices, San Jose, CA, USA). Based on a calibration curve generated from the six standards mentioned above, AFM1 concentrations were determined using RIDASOFT^®^ Win.NET software (version 1.98; R-Biopharm AG, Darmstadt, Germany).

The performance of the kit was evaluated by spiking uncontaminated milk with AFM1 at concentrations of 10, 20, and 40 ng/L (in triplicate), followed by quantification of the spiked samples. The recovery rates ranged from 88.80% to 102.56%, with coefficients of variation between 2.19% and 5.59% (Supplementary Table S1). These results meet the performance criteria for the kit set by Chinese authorities [17]. Samples with AFM1 readings above the highest spiked concentration (40 ng/L) were diluted appropriately with the kit-provided dilution buffer and re-analyzed; the final concentration was calculated by applying the dilution factor.

### 2.3. Assessment of AFM1 Exposure and Associated Health Risks

#### 2.3.1. AFM1 Exposure Assessment

The estimated daily intake (EDI) of AFM1 for milk consumers was calculated using a deterministic approach recommended by the International Programme on Chemical Safety [18], as follows:

(1) EDI (ng/kg bw/day) = (C × V)/W

where C represents the mean AFM1 concentration in milk (ng/L), V denotes daily milk consumption (L), and W is the mean body weight (kg) of the target population. For samples with no detection of AFM1 (<LOD), the AFM1 content was conservatively assumed as 50% of the LOD (i.e., 2.5 ng/L). For this assessment, daily milk intake was set at four levels (100, 200, 300, and 400 mL) based on Chinese consumption data (mean, 126 mL/day; 95th percentile, 340 mL/day) [13]. The body weight data were derived from the national physical fitness survey [19].

#### 2.3.2. Health Risks from AFM1 Exposure

- MOE-based Calculation of AFM1 in milk

Given the genotoxic and carcinogenic properties of AFM1, an additional MOE approach was employed for quantitative risk characterization, following the guidelines established by the European Food Safety Authority (EFSA) for substances with such attributes [4]. The MOE was calculated as the ratio of the benchmark dose lower confidence limit for a 10% response (BMDL_10_) to the EDI of AFM1, as follows:

(2) MOE = BMDL_10_/EDI

where BMDL_10_ represents the benchmark dose lower confidence limit associated with a 10% increased risk of cancer. Since specific BMDL_10_ data for AFM1-induced hepatocellular carcinoma (HCC) are unavailable, a potency factor of 0.1 relative to AFB1 was applied to extrapolate the BMDL_10_ from the established AFB1 consumption rate of 400 ng/kg bw/day [4], yielding a BMDL_10_ of 4000 ng/kg bw/day for AFM1. The EDI was obtained from previous detailed exposure assessments, representing the EDI of AFM1 via milk consumption across different population groups. Risk interpretation was performed in accordance with the EFSA guidelines, where MOE ≥ 10,000 was deemed indicative of low public health concern regarding AFM1 exposure and MOE < 10,000 signified a potential health risk associated with such exposure [4].

- Quantitative calculation of AFM1-induced HCC risk

The population risk of HCC associated with dietary AFM1 exposure was quantified using a dose–response approach based on the carcinogenic potency factors established by the Joint FAO/WHO Expert Committee on Food Additives (JECFA), accounting for AFB1 consumption and synergistic effects with HBV infection [3]. Given that AFM1 exhibits approximately one-tenth the carcinogenic potency of AFB1 [3], the cancer potency values were derived by scaling established potency estimates accordingly. Thus, for HBV surface antigen-positive (HBsAg+) individuals, the carcinogenic potency of AFM1 was set at 0.0562 additional cases per 100,000 persons per year per 1 ng/kg bw/day, while for HBsAg− individuals, the corresponding value was 0.0049 [3]. To account for the prevalence of HBV in the study population, the average carcinogenic potency across the different age-sex cohorts in northwest and south–central China was calculated as follows:

(3) Average potency = (HBsAg+ potency × P) + HBsAg− potency × (1 − P)

where P represents the regional HBsAg+ prevalence rate (3.2% and 5.0% for northwest and south–central China, respectively; [20]). Subsequently, the excess HCC risk attributed to AFM1 exposure was then determined by multiplying the AFM1 EDI by the population-specific average potency, as follows:

(4) Cancer risk = EDI × Average potency

This calculation yields the estimated number of additional HCC cases per 100,000 persons per year associated with AFM1 exposure through milk consumption in the target population.

### 2.4. Statistical Analysis

For all samples, the AFM1 positivity rate and the percentage exceeding the legal limits of both the EU and China were determined. For AFM1-positive samples, the concentration was further characterized as the median, 95th percentile, maximum, and mean ± SD. The effects of milk type, sampling season, and geographic region on milk AFM1 levels were analyzed separately using the non-parametric Mann–Whitney U test with IBM SPSS Statistics for Windows (version 19.0; IBM Corp., Armonk, NY, USA). The threshold for statistical significance was set at *p* < 0.05.

## 3. Results

### 3.1. Occurrence and Seasonal Variation in AFM1 in Milk Products

**Figure 1:**
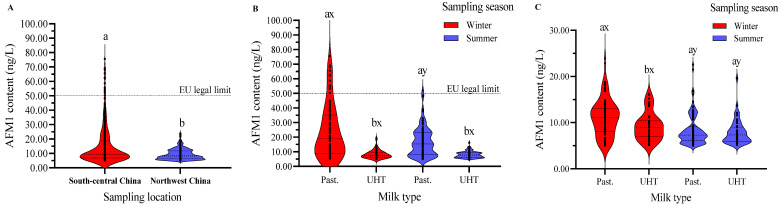
Comparison of AFM1-positive milk samples. (**A**): Comparison of AFM1-positive milk samples collected in south–central and northwest China. (**B**): Comparison of AFM1-positive milk samples collected in south–central China. (**C**): Comparison of AFM1-positive milk samples collected in northwest China. Note: (1) The violin plot displays the median (dotted line), interquartile range (dashed line), and EU legal limit (dash-dotted line). (2) In (**A**), different letters (a, b) indicate significant differences between groups (*p* < 0.05). (3) In (**B**,**C**), different letters (a, b) indicate significant differences between groups during the same season (*p* < 0.05); different letters (x, y) indicate significant differences between groups of the same milk type (*p* < 0.05). (4) UHT: ultra-high temperature milk; Past.: pasteurized milk.

Of the 902 milk samples analyzed, 680 (75.39%) tested positive for AFM1. Among these positive samples, the median, 95th percentile, maximum, and mean ± SD concentrations were 8.96, 32.78, 75.57, and 12.35 ± 10.27 ng/L, respectively (Table 1). Notably, 14 samples (1.55% of the total) exceeded the EU ML of 50 ng/L, and all were pasteurized milk. All samples, however, were compliant with the China ML of 500 ng/L. Geographically, median AFM1 levels were significantly higher in milk samples from south–central China than northwest China (9.40 vs. 8.30 ng/L, respectively, *p* < 0.05; Figure 1A). Pasteurized milk from south–central China during winter had the most severe contamination, with median, 95th percentile, and maximum values of 19.22, 65.34, and 75.57 ng/kg, respectively (Table 1). Seasonal analysis revealed that the median concentrations of AFM1 in positive samples were higher in winter than summer in both regions (Figure 1B,C). AFM1 levels in northwest China were 38.46% higher in winter than summer, with both milk types showing a significant increase of 1.22 to 1.53 fold (*p* < 0.05). However, AFM1 levels in milk from south–central China exhibited moderate seasonal variation (7.10% overall increase), where pasteurized milk demonstrated a significant 1.25-fold elevation in winter (*p* < 0.05), while levels in UHT milk remained stable (*p* > 0.05). Regarding milk types, median AFM1 concentrations were higher in pasteurized milk than UHT milk. This difference was significant in south–central China during both seasons (1.98–2.56 times higher, *p* < 0.05) and in northwest China during winter (1.24 times higher, *p* < 0.05), but not during summer in the northwest (*p* > 0.05). Overall, winter pasteurized milk from south–central China had the most severe AFM1 contamination, with some samples exceeding the EU limit, while summer UHT milk from northwest China demonstrated the lowest AFM1 levels, as all samples were far below the China ML.

### 3.2. Population Exposure to AFM1 in Milk

The EDI of AFM1 was significantly associated with age, geographical region, and milk consumption volume. Preschool children (age, 3–6 years) exhibited the highest exposure levels, particularly in south–central China where 400 mL/day consumption resulted in EDIs of 0.261 and 0.273 ng/kg bw/day for males and females, respectively. Across all age groups, females consistently exhibited numerically higher EDIs than males (2.22–25.00% difference). After controlling for sex and milk intake, milk from south–central China had 1.33–1.54 times more AFM1 than milk from northwest China. Moreover, a clear dose–response relationship was observed, demonstrating an approximate 4.00-fold increase in EDI as consumption rose from 100 to 400 mL/day. Heavy consumers (≥400 mL/day) in south–central China, especially preschoolers, reached exposure levels, thereby warranting attention (0.2 ng/kg bw/day), while all other groups remained below this safety threshold (Table 2). Therefore, to control AFM1 intake, milk consumers need to comprehensively consider influencing factors, such as age, region, and consumption volume.

### 3.3. AFM1 Risk Characterization

#### 3.3.1. MOE-Based Risk Characterization of AFM1 in Milk

**Figure 2:**
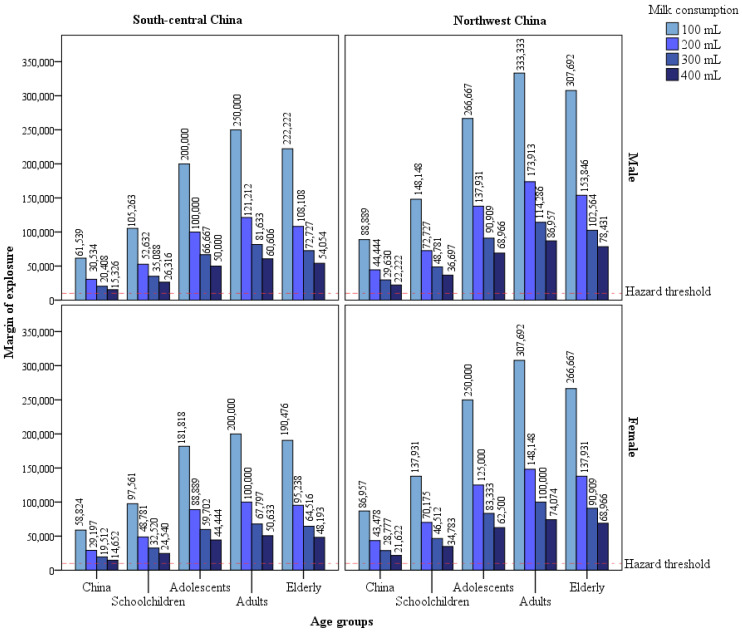
Margin of exposure (MOE) for AFM1 exposure across different age-sex groups by milk intake in south–central and northwestern China. An MOE value > 10,000 indicates a potential health risk.

Analysis of MOE values demonstrated substantial variation in exposure risk across population groups and geographical regions, as all values remained above the hazard threshold of 10,000 (Figure 2). Preschool children (age, 3–6 years) in south–central China had the highest exposure risk, with MOE values approaching the hazard threshold of 10,000 at 400 mL/day consumption (girls, 14,652; boys, 15,326). MOE values showed an age-dependent increase from preschoolers to adults followed by a modest decline in elderly populations. Milk consumers in south–central China had systematically lower MOE values compared to those in northwest China at equivalent intake levels, with the values 1.38 to 1.48 times higher in the northwest. Therefore, based on the MOE assessment, all milk consumers remain within safe limits. Nevertheless, from the perspective of AFM1 control in milk, region-specific monitoring strategies are warranted, particularly where vulnerable preschool-aged children with high milk consumption reside.

#### 3.3.2. HCC Risk Characterization of AFM1 in Milk

The quantitative risk assessment identified distinct geographical and age-related variations in AFM1-attributable HCC risk across the studied populations (Table 3). South–central China demonstrated consistently higher population-level risks than northwest China across all age groups, with milk consumers facing 44.44–70.00% higher carcinogenic risks. Preschool children (age, 3–6 years) exhibited the most elevated risk levels relative to other age groups. At consumption of 400 mL/day, the potential risk of liver cancer for preschoolers in south–central China was 0.00204 cases per 100,000 person-years for girls and 0.00195 for boys. These values were approximately 1.69 times higher than those of their counterparts in northwest China. Interestingly, the risk decreased progressively from preschoolers to adults, with the lowest risk found in adults, and then rebounded slightly in the elderly (age, ≥60 years). Yet, sex-based disparities were not pronounced, at only about 4.62%. Consequently, high milk consumers in south–central China, particularly preschool children, face the greatest potential liver cancer risk, calling for targeted interventions and strengthened controls.

## 4. Discussion

### 4.1. Prevalence of AFM1 in Milk

AFM1 contamination of milk remains a key safety concern in China. This study assessed geographic, seasonal, and milk-type variations in AFM1 contamination of milk to reveal the contamination characteristics.

Our investigations showed higher AFM1 contamination levels in south–central China than northwest China. Consistent with our findings, Li et al. [21] reported higher AFM1 concentrations in milk samples from central China as compared to the northwest region (46.7 vs. 19.9 ng/L, respectively). These regional differences in milk AFM1 contamination have been associated with variations in climate and feeding practices. Northwestern China has a temperate continental climate, with a mean temperature of 20.2 °C in summer and −4.7 °C in winter, a mean rainfall of 35.4 mm/month in summer and 2.1 mm/month in winter, and a mean relative humidity of 59.0% in summer and 48.8% in winter. In contrast, south–central China experiences a subtropical humid monsoon climate, with corresponding means of 26.5 °C in summer and 6.8 °C in winter, rainfall of 126.2 mm/month in summer and 60.5 mm/month in winter, and relative humidity of 78.2% in summer and 76.4% in winter. (climate data source: https://data.cma.cn/, accessed on 5 January 2026; Supplementary Table S4). This warmer and more humid environment better supports fungal proliferation and AFB1 biosynthesis in feed, as higher temperatures and elevated water activity have been shown to promote *A. flavus* growth and aflatoxin production [22,23], ultimately leading to more severe AFM1 contamination of milk from south–central China. AFM1 levels in milk were far below the legal limit in China, indicating effective control measures by regulatory authorities, dairy industries, and farms in these regions. However, the high AFM1 detection rates in both surveyed regions—with south–central China posing elevated risks of exceeding EU limits—highlight the need for improved feed storage practices and strengthened monitoring of AFB1 in feed and AFM1 in milk, particularly in south–central China.

This study found generally higher AFM1 contamination in winter than summer, aligning with previous studies [1,24,25]. Consistent with our findings, a study conducted in Iran reported higher mean AFM1 levels in winter than summer (55.28 vs. 30.33 ng/L, respectively; [26]). Similarly, a study performed in the Middle East also found higher AFM1 positivity rates in winter (87% vs. 70.6%, respectively; [27]). These findings reinforce seasonal patterns in AFM1 contamination of milk. The higher AFM1 contamination of milk in winter primarily results from reduced availability of fresh pasture, which leads to greater reliance on stored feeds, such as silage, corn, peanut meal, and concentrates. These feeds are more prone to AFB1 contamination, thereby increasing the transfer of AFM1 into milk [13,26,28]. Furthermore, regional climate also plays a key role in the seasonal dynamics of AFM1 contamination, as levels are typically higher in subtropical humid monsoon areas than temperate arid zones [13]. Consequently, implementing routine toxin screening during winter is crucial, especially in humid regions.

Our study indicated that pasteurized milk contained higher AFM1 concentrations than UHT milk. This finding aligns with a study conducted in central-eastern China, which reported significantly higher AFM1 levels in pasteurized milk than UHT milk (18.6 vs. 11.9 ng/L, respectively) [15]. Another study conducted in central China also reported significantly higher AFM1 levels in pasteurized milk than UHT milk, with concentrations of 32.6 vs. 10.1 ng/L in winter and 15.2 vs. 9.7 ng/L in summer, respectively [25]. AFM1 contamination of liquid milk is influenced by the raw milk source, processing method, and regulatory oversight. Pasteurized and UHT milk often involve pooling from multiple sources, thereby increasing the risk of AFM1 contamination if not strictly monitored [29]. Stringent raw milk selection combined with centralized high-volume mixing procedures in UHT production systems may reduce AFM1 levels in milk [30,31], whereas the higher AFM1 levels in pasteurized milk may be associated with localized, decentralized production and small-scale batch processing methods [30]. Furthermore, the differences in AFM1 levels between UHT and pasteurized milk may stem from distinct temperature-time processing parameters. A previous study indicated that pasteurization did not alter AFM1 levels in milk, as high-temperature treatments, such as boiling and sterilization, effectively reduced the AFM1 content by 15.79% and 21.74%, respectively [32]. However, Awasthi et al. [33] found that neither pasteurization nor boiling significantly altered milk AFM1 levels. Those studies have reported variable efficacy of heat treatments to degrade AFM1 in milk. Thus, stringent quality control of raw milk and effective batch management are critical to combat AFM1 contamination of liquid dairy products. In addition to monitoring and quality management methods, several technical measures can help mitigate AFM1 in milk, including non-thermal processing, such as ozonation, ultraviolet irradiation, pulsed electric fields [34] and liquid-phase plasma [35], as well as emerging biological detoxification methods using yeasts and lactic acid bacteria [36], enzymes [37], and metal–organic-framework-based composite membranes [38].

Overall, to prevent AFM1 contamination of milk, comprehensive measures—such as feeding management and AFM1 monitoring—should be implemented, while accounting for the key factors of region, season, and milk type.

### 4.2. Risk of Exposure to AFM1 for Milk Consumers

This study systematically evaluated the risk of AFM1 exposure to milk consumers through multiple assessment metrics, including EDI, MOE, and PRLC. Among the five age groups surveyed, preschool children (age, 3–6 years) had the highest EDI values, which decreased with age to the lowest level in adults before rebounding slightly in the elderly population, consistent with the findings of Bilandžić et al. [1] and Xiong et al. [15]. This observed trend may be related to variations in milk consumption per unit body weight across age groups. Preschool children face significantly higher risks of AFM1 exposure as compared to other age groups, as documented by studies conducted in China (Table 4), Croatia [1], India [39], and Mexico [40]. The highest EDI values of children in south–central China were comparable to those reported in central-eastern China (EDI, 0.23–0.25 ng/kg bw/day) [15]. To mitigate exposure to preschoolers, milk AFM1 levels should be minimized. Moreover, the risk of AFM1 exposure for preschool children was lower in northwest China than south–central regions, with the EDI values higher than that in the Xinjiang Uygur autonomous region of China (0.030–0.053 ng/kg bw/day) [12]. Consequently, AFM1 exposure risks in children require heightened attention across both surveyed regions, particularly in south–central China. There were no substantial differences in the EDI values of AFM1 between male and female milk consumers, consistent with prior studies [1,15,41]. Therefore, sex-specific measures are unwarranted for managing non-carcinogenic risks from AFM1, as increased consumption of milk and dairy products is the key driver of exposure risk [42].

The MOE is used to assess the long-term carcinogenic risk of AFM1, with values < 10,000 considered to indicate potential risk [4]. All population groups in this study exhibited MOE values exceeding 10,000, indicating no carcinogenic risk at the present consumption of 100–400 mL/day. This finding is consistent with those reported in other Chinese studies (see Table 4 for references). However, Dong et al. [45], who calculated upper-bound AFM1 levels by substituting non-detects with detection limits, derived an MOE of 7057 for child consumers in Shandong province, China. While this method may overestimate actual exposure, continued attention is warranted to reduce AFM1 exposure risks. Furthermore, preschool populations in south–central China, with milk consumption exceeding 586 mL/day for females and 613 mL/day for males, would pose a risk based on an MOE value < 10,000. Thus, milk consumers in the surveyed regions were at negligible carcinogenic risk from AFM1 exposure at the present consumption levels. However, preschoolers in south–central China with higher milk consumption warrant vigilance against long-term carcinogenic risk.

This study revealed the highest PRLC in preschoolers, lowest in adults, with a slight rebound in the elderly. At a daily intake of 400 mL, the HCC risk for preschoolers (0.00118–0.00204 cases per 100,000 person-years) was higher than reported for Chinese children (0.00004–0.00012; [13]), but lower than the risk estimated for Albanian children (0.021; [47]). These differences were closely related to the prevalence of HBV, milk consumption, and the rate of AFM1 contamination of milk [3]. Based on the reported crude incidence rate of liver cancer in China (26.04 cases/100,000 person-years) [48], AFM1-related liver cancer risk in preschoolers contributes very little (0.001–0.008%) to the total HCC burden. However, higher milk consumers were at elevated risks with MOE values < 10,000, warranting prioritized surveillance for this subgroup.

Although the risk assessment in this study indicates that preschoolers face non-carcinogenic risks from AFM1 contamination of milk, the cumulative carcinogenic potential warrants attention, especially under conditions of high milk consumption. As milk intake amounts continues to rise among the population, ongoing assessment of exposure risks is necessary to provide references for safe milk consumption. Given the strong carcinogenicity of AFM1, the following measures are recommended to reduce the incidence of milk contamination: enhanced monitoring and decontamination of AFB1 in feed to lower milk contamination; stricter controls of milk AFM1 (applying the “as low as reasonably achievable” principle) for high-risk groups/regions; and balanced nutrition strategies that include optimized milk sourcing, promotion of low-AFM1 dairy products, and intake guidance.

In this large-scale survey, we used a commercially available ELISA kit, which is widely accepted for screening large numbers of samples due to its high throughput, cost-effectiveness, and good sensitivity. However, the ELISA method used for AFM1 quantification is a screening method, which is a limitation of this study, as matrix effects and cross-reactivity may introduce bias. Although the kit was validated with acceptable recovery and linearity, and all samples were analyzed in duplicate, confirmatory analysis by more precise methods such as HPLC or LC-MS/MS was not performed. In fact, highly sensitive LC-MS/MS methods have been established for AFM1 analysis, including simplified AFM1 protocols [49], sensitivity-enhanced LC-IDMS [50], and a QuEChERS-based multi-toxin LC-MS/MS method [51]. Future studies on milk AFM1 contamination should consider adopting these advanced LC-MS/MS techniques to achieve the highest confidence in quantitative results, especially when the data are intended for regulatory or refined risk assessment purposes. Nonetheless, the clear seasonal, regional, and milk-type differences in AFM1 contamination observed in this study, together with its good agreement with previous studies [1,13,52], support the overall validity of our conclusions.

## 5. Conclusions

This study revealed that contamination levels were higher in south–central China than northwest China, in winter than summer, and in pasteurized milk than UHT milk, with the most severe contamination observed in pasteurized milk from south–central China during winter. All milk samples complied with the China ML, although 1.6% exceeded the EU ML. Preschoolers in south–central China consuming 400 mL/day of milk are the group with the highest AFM1 exposure risk. The surveyed population groups were at negligible carcinogenic risk from AFM1 exposure, as assessed by MOE and PRLC values. Therefore, differentiated management strategies based on region, season, and processing type are recommended, with priority given to targeted monitoring and dietary guidance for high-risk preschooler populations.

## Figures and Tables

**Table 1:** Occurrence of AFM1 in pasteurized and UHT milk samples from northwest and south–central China during summer and winter.

Area	Sampling Season	Sample Type ^a^	Positives/Total Sample No. ^b^ (%)	Exceeding EU ML ^c^ of 50 ng/L No. (%)	Exceeding China ML of 500 ng/L No. (%)	AFM1 Concentration in Positive Samples (ng/L)			
						Median	P95 ^d^	Max.	Mean ± SD
Northwest China	Summer	Pasteurized	112/133 (84.21)	0	0	7.28	13.96	22.58	8.36 ± 3.30
		UHT	44/75 (58.67)	0	0	7.33	12.11	19.56	7.98 ± 2.76
	Subtotal summer milk		156/208 (75.00)	0	0	7.28	13.55	22.58	8.25 ± 3.16
	Winter	Pasteurized	105/120 (87.50)	0	0	11.11	18.02	23.85	11.05 ± 3.96
		UHT	56/81 (69.14)	0	0	8.97	15.20	16.19	9.24 ± 2.99
	Subtotal winter milk		161/201 (80.10)	0	0	10.08	17.27	23.85	10.42 ± 3.74
Total milk for northwest China			317/409 (77.51)	0	0	8.30	16.74	23.85	9.35 ± 3.63
South–central China	Summer	Pasteurized	99/133 (74.44)	1 (0.75)	0	15.36	33.49	52.08	16.78 ± 10.17
		UHT	93/126 (73.81)	0	0	7.76	11.72	16.17	8.03 ± 2.19
	Subtotal summer milk		192/259 (74.13)	1 (0.39)	0	9.15	29.97	52.08	12.54 ± 8.64
	Winter	Pasteurized	102/107 (95.33)	13 (12.15)	0	19.22	65.34	75.57	24.07 ± 18.53
		UHT	69/127 (54.33)	0	0	7.52	13.02	19.01	8.27 ± 2.83
	Subtotal winter milk		171/234 (73.08)	13 (5.56)	0	9.80	57.08	75.57	17.70 ± 16.36
Total milk for south–central China			363/493 (73.63)	14 (2.84)	0	9.40	42.45	75.57	14.97 ± 13.10
TOTAL milk			680/902 (75.39)	14 (1.55)	0	8.96	32.78	75.57	12.35 ± 10.27

^a^ UHT: ultra-high temperature milk. ^b^ Positive samples are defined as those in which the AFM1 concentration exceeded the limit of detection of 5 ng/L. ^c^ ML: maximum level. ^d^ P95 represents the 95th percentile value. The AFM1 levels and detailed information for individual samples are provided in Supplementary Tables S2 and S3.

**Table 2:** Variation in the EDI values of AFM1 by age-sex groups and milk consumption level in two surveyed regions.

Age Groups	Median Weight (kg) ^a^				Milk Consumption (mL) ^b^	Median EDI (ng/kg bw/Day) ^c^			
	South–Central China		Northwest China			South–Central China		Northwest China	
	Male	Female	Male	Female		Male	Female	Male	Female
Preschoolers (3–6 years)	17.9	17.1	17.4	16.9	100	0.065	0.068	0.045	0.046
					200	0.131	0.137	0.090	0.092
					300	0.196	0.205	0.135	0.139
					400	0.261	0.273	0.180	0.185
School-aged children (6–12 years)	30.8	28.6	28.6	27.2	100	0.038	0.041	0.027	0.029
					200	0.076	0.082	0.055	0.057
					300	0.114	0.123	0.082	0.086
					400	0.152	0.163	0.109	0.115
Adolescents (12–18 years)	58.4	52.1	53.8	49.0	100	0.020	0.022	0.015	0.016
					200	0.040	0.045	0.029	0.032
					300	0.060	0.067	0.044	0.048
					400	0.080	0.090	0.058	0.064
Adults (18–60 years)	70.8	59.1	67.9	57.9	100	0.016	0.020	0.012	0.013
					200	0.033	0.040	0.023	0.027
					300	0.049	0.059	0.035	0.040
					400	0.066	0.079	0.046	0.054
Elderly (≥60 years)	63.4	56.2	60.7	53.6	100	0.018	0.021	0.013	0.015
					200	0.037	0.042	0.026	0.029
					300	0.055	0.062	0.039	0.044
					400	0.074	0.083	0.051	0.058
ALL	17.9–70.8	17.1–59.1	59.5	50.9	100–400	0.016–0.261	0.020–0.273	0.012–0.180	0.013–0.185

Note: “Heat map” (scale: green-yellow-red color levels) reflects EDI levels of AFM1 exposure for different age–gender groups at milk consumption levels of 100–400 mL in central and northwest China. ^a^ The data were calculated based on the dataset reported by Zhao, Ding, and Zhao [19]. ^b^ Daily milk consumption levels (100, 200, 300, and 400 mL) were selected for exposure assessment, referencing reported Chinese consumption data (mean: 126 mL/day; 95th percentile: 340 mL/day) [13]. ^c^ The EDI values were calculated based on mean regional AFM1 concentrations (Northwest China: 7.81 ng/L; South–Central China: 11.68 ng/L), derived by assigning negative samples a value of 2.5 ng/L (half the 5.0 ng/L detection limit).

**Table 3:** Estimated risk of liver cancer from AFM1 exposure by age-sex groups and milk consumption level in two surveyed regions.

Age Groups	Milk Consumption (mL) ^a^	Liver Cancer Risk from Milk AFM1 Intake (Cases/100,000 Persons/Year) ^b^			
		South–Central China		Northwest China	
		Male	Female	Male	Female
Preschoolers (3–6 years)	100	0.00049	0.00051	0.00029	0.00030
	200	0.00098	0.00102	0.00059	0.00060
	300	0.00146	0.00153	0.00088	0.00091
	400	0.00195	0.00204	0.00118	0.00121
School-aged children (6–12 years)	100	0.00028	0.00031	0.00018	0.00019
	200	0.00057	0.00061	0.00036	0.00037
	300	0.00085	0.00092	0.00054	0.00056
	400	0.00114	0.00122	0.00071	0.00075
Adolescents (12–18 years)	100	0.00015	0.00016	0.00010	0.00010
	200	0.00030	0.00034	0.00019	0.00021
	300	0.00045	0.00050	0.00029	0.00031
	400	0.00060	0.00067	0.00038	0.00042
Adults (18–60 years)	100	0.00012	0.00015	0.00008	0.00009
	200	0.00025	0.00030	0.00015	0.00018
	300	0.00037	0.00044	0.00023	0.00026
	400	0.00049	0.00059	0.00030	0.00035
Elderly (≥60 years)	100	0.00013	0.00016	0.00009	0.00010
	200	0.00028	0.00031	0.00017	0.00019
	300	0.00041	0.00046	0.00026	0.00029
	400	0.00055	0.00062	0.00033	0.00038

Note: “Heat map” (scale: green-yellow-red color levels) reflecting liver cancer risk of AFM1 exposure for different groups of milk consumers in south–central and northwest China. ^a^ Daily milk consumption levels (100, 200, 300, and 400 mL) were selected for exposure assessment, referencing reported Chinese consumption data (mean: 126 mL/day; 95th percentile: 340 mL/day) [13]. ^b^ Liver cancer risk was calculated using two regional parameters: (1) mean AFM1 concentrations (South–Central China: 7.81 ng/L; Northwest China: 11.68 ng/L), with non-detect samples assigned a value of 2.5 ng/L (half the detection limit); and (2) HBsAg+ prevalence rates (South–Central China: 5.0%; Northwest China: 3.2%), as reported by Yan et al. [20].

**Table 4:** Review of AFM1 risk characterization in three age groups of male and female milk consumers in China (2013–2025).

Age Group	Gender	Milk Type	ED I ^a^ (ng/kg b.w./d)	MOE ^b^	HCC ^c^ Risk (×10^−6^ Cases/100,000 Persons/Year)	Province	Year	Ref.
Children (2~7 years)	Male	Milk	0.1055–0.1612	24,814–37,915	325.1–496.8	Shandong	2025	[43]
	Female	Milk	0.1010–0.1515	26,403–39,604	311.2–466.9	Shandong	2025	[43]
	Both	Milk	0.0367–0.0730	54,795–108,992	110–220	25 provinces and municipalities	2025	[13]
	Both	Milk	0.0026–0.5668	7057–1,538,462	/^d^–2500	Shandong	2023	[44]
	Male	Milk, Yogurt	0.16–0.25	16,000–25,000	/	Hubei, Zhejiang, and Fujian	2022	[15]
	Female	Milk, yogurt	0.16–0.23	17,391–25,000	/	Hubei, Zhejiang, and Fujian	2022	[15]
	Male	Milk	0.032–0.053	75,472–125,000	/	Xinjiang	2022	[12]
	Female	Milk	0.030–0.050	80,000–133,333	/	Xinjiang	2022	[12]
	Male	Milk	0.178–0.272	14,706–22,472	487–745	Shaanxi	2020	[45]
	Female	Milk	0.170–0.255	15,686–23,529	467–700	Shaanxi	2020	[45]
	Male	Milk, yogurt	0.034–0.079	50,633–117,647	/	31 provinces	2013	[46]
	Female	Milk, yogurt	0.030–0.087	45,977–133,333	/	31 provinces	2013	[46]
**Subtotal**	**Male,** **female and both**	**Milk, yogurt**	**0.0026–0.5668**	**7057–1,538,462**	**110–2500**	**31 provinces**	**2013–2025**	**7 references**
Adult (18~60 years)	Male	Milk	0.0245–0.0271	147,601–163,265	753–837	Shandong	2025	[43]
	Female	Milk	0.0273–0.0326	122,624–146,520	840–1005	Shandong	2025	[43]
	Both	Milk	0.0076–0.0161	248,447–526,316	20–50	25 provinces and municipalities	2025	[13]
	Male	Milk, yogurt	0.04–0.05	80,000–100,000	/	Hubei, Zhejiang, and Fujian	2022	[15]
	Female	Milk, yogurt	0.04–0.05	80,000–100,000	/	Hubei, Zhejiang, and Fujian	2022	[15]
	Male	Milk	0.008–0.009	444,444–500,000	/	Xinjiang	2022	[12]
	Female	Milk	0.009–0.011	363,636–444,444	/	Xinjiang	2022	[12]
	Male	Milk	0.041–0.046	86,957–97,561	113–125	Shaanxi	2020	[45]
	Female	Milk	0.046–0.055	72,727–86,957	126–151	Shaanxi	2020	[45]
	Male	Milk, yogurt	0.005–0.008	500,000–800,000	/	31 provinces and municipalities in China	2013	[46]
	Female	Milk, yogurt	0.006–0.009	444,444–666,667	/	31 provinces and municipalities	2013	[46]
**Subtotal**	**Male, female and both**	**Milk, yogurt**	**0.005–0.055**	**72,727–800,000**	**20–1005**	**31 provinces**	**2013–2025**	**6 references**
Elderly (≥60 years)	Male	Milk	0.0306– 0.0360	111,111–130,719	943–1111	Shandong	2025	[43]
	Female	Milk	0.0330–0.0397	100,756–121,212	1018–1224	Shandong	2025	[43]
	Both	Milk	0.0091–0.0192	208,333–439,560	30–60	25 provinces and municipalities	2025	[13]
	Male	Milk, yogurt	0.05–0.06	66,667–80,000	/	Hubei, Zhejiang, and Fujian	2022	[15]
	Female	Milk, yogurt	0.05–0.06	66,667–80,000	/	Hubei, Zhejiang, and Fujian	2022	[15]
	Male	Milk	0.010–0.012	333,333–400,000	/	Xinjiang	2022	[12]
	Female	Milk	0.010–0.013	307,692–400,400	/	Xinjiang	2022	[12]
	Male	Milk	0.052–0.061	65,574–76,923	141–167	Shaanxi	2020	[45]
	Female	Milk	0.056–0.067	59,701–71,429	153–184	Shaanxi	2020	[45]
	Male	Milk, yogurt	0.011–0.012	333,333–363,636	/	31 provinces and municipalities	2013	[46]
	Female	Milk, yogurt	0.011–0.012	333,333–363,636	/	31 provinces and municipalities	2013	[46]
**Subtotal**	**Male,** **female and both**	**Milk, yogurt**	**0.0091–0.067**	**59,701–439,560**	**30–1224**	**31 provinces and municipalities**	**2013–2025**	**6 references**
**TOTAL**	**Male, female** **and both**		**0.0026–0.5668**	**7057–1,538,462**	**20–2500**	**31 provinces and municipalities**	**2013–2025**	**7 references**

^a^ EDI: estimated daily intake. ^b^ MOE: margin of exposure. MOE is calculated using the following equation: MOE = BMDL_10_/EDI, where BMDL_10_ (the benchmark dose lower confidence limit for a 10% response) was set to 4000 [4]. ^c^ HCC: hepatocellular carcinoma. ^d^ Unavailable in the reference.

## Data Availability

The original contributions presented in the study are included in the article/Supplementary Material, further inquiries can be directed to the corresponding authors.

## Supplementary Materials

The following supporting information can be downloaded at: https://www.mdpi.com/article/10.3390/foods15122102/s1, Table S1: Validation of AFM1 detection in milk; Table S2: AFM1 concentration in two types of milk from northwest China; Table S3: AFM1 concentration in two types of milk from south–central China; Table S4: Temperature and humidity in the two regions during the summer and winter surveys.
