# Supplementary material for: Aflatoxin M1 in Milk from South–Central and Northwest China: Prevalence and Integrated Risk Characterization for Different Age-Sex Groups of Consumers
Source: Foods. 2026 Jun 11;15(12):2102. doi: 10.3390/foods15122102 (PMC13298350; doi:10.3390/foods15122102)
Supplement: Supplementary file 1 [file foods-15-02102-s001.zip › foods-4337862-supplementary.pdf]

Supplementary materials

Table S1. Validation of AFM1 detection in milk.

AFM1 spiked (ng/L)	AFM1 found (ng/L)	Mean ± SD (ng/L)	Recovery (%)	Coefficient of variation (%)
10	8.31	8.88 ± 0.50	88.80	5.59
	9.22			
	9.11			
20	18.41	19.23 ± 0.74	96.17	3.83
	19.83			
	19.46			
40	42.04	41.02 ± 0.90	102.56	2.19
	40.70			
	40.33			

Table S2. AFM1 concentration in two types of milk from northwest China.

Serial Number	Sample ID	Milk type	Region (province) sampled	Season	AFM1 concentration (ng/L)
1	D5-0818	Pasteurized	Northwest China (GS)	Summer	12.16
2	D5-0819	Pasteurized	Northwest China (GS)	Summer	7.20
3	D5-0820	Pasteurized	Northwest China (GS)	Summer	5.39
4	D5-0821	Pasteurized	Northwest China (GS)	Summer	5.14
5	D5-0822	Pasteurized	Northwest China (GS)	Summer	6.97
6	D5-0823	Pasteurized	Northwest China (GS)	Summer	5.56
7	D5-0824	Pasteurized	Northwest China (GS)	Summer	7.74
8	D5-0825	Pasteurized	Northwest China (GS)	Summer	5.45
9	D5-0826	Pasteurized	Northwest China (GS)	Summer	8.06
10	D5-0828	Pasteurized	Northwest China (GS)	Summer	5.43
11	D5-0829	Pasteurized	Northwest China (GS)	Summer	<5
12	D5-0830	Pasteurized	Northwest China (GS)	Summer	5.79
13	D5-0831	Pasteurized	Northwest China (GS)	Summer	8.08
14	D5-0901	Pasteurized	Northwest China (GS)	Summer	5.53
15	D5-0902	Pasteurized	Northwest China (GS)	Summer	6.21
16	D5-0903	Pasteurized	Northwest China (GS)	Summer	<5
17	D5-0904	Pasteurized	Northwest China (GS)	Summer	7.36
18	D5-0905	Pasteurized	Northwest China (GS)	Summer	8.15
19	D5-0906	Pasteurized	Northwest China (GS)	Summer	7.65
20	D5-0907	Pasteurized	Northwest China (GS)	Summer	12.44
21	D5-0908	Pasteurized	Northwest China (GS)	Summer	8.27
22	D5-0909	Pasteurized	Northwest China (GS)	Summer	7.18
23	D5-0910	Pasteurized	Northwest China (GS)	Summer	7.57
24	D5-0911	Pasteurized	Northwest China (GS)	Summer	7.26

Serial Number	Sample ID	Milk type	Region (province) sampled	Season	AFM1 concentration (ng/L)
25	D5-0912	Pasteurized	Northwest China (GS)	Summer	6.80
26	D5-0913	Pasteurized	Northwest China (GS)	Summer	5.40
27	D5-0914	Pasteurized	Northwest China (GS)	Summer	6.68
28	D5-0915	Pasteurized	Northwest China (GS)	Summer	7.65
29	D5-0916	Pasteurized	Northwest China (GS)	Summer	6.95
30	D5-0917	Pasteurized	Northwest China (GS)	Summer	7.27
31	D5-0918	Pasteurized	Northwest China (GS)	Summer	8.25
32	D5-0919	Pasteurized	Northwest China (GS)	Summer	7.15
33	D5-0920	Pasteurized	Northwest China (GS)	Summer	6.14
34	D5-0921	Pasteurized	Northwest China (GS)	Summer	5.19
35	D5-0922	Pasteurized	Northwest China (GS)	Summer	7.17
36	D5-0923	Pasteurized	Northwest China (GS)	Summer	<5
37	D5-0924	Pasteurized	Northwest China (GS)	Summer	<5
38	D5-0925	Pasteurized	Northwest China (GS)	Summer	5.95
39	D5-0926	Pasteurized	Northwest China (GS)	Summer	8.10
40	D5-0927	Pasteurized	Northwest China (GS)	Summer	8.59
41	Y7-0818	Pasteurized	Northwest China (GS)	Summer	10.84
42	Y7-0820	Pasteurized	Northwest China (GS)	Summer	<5
43	Y7-0821	Pasteurized	Northwest China (GS)	Summer	5.87
44	Y7-0822	Pasteurized	Northwest China (GS)	Summer	5.42
45	Y7-0823	Pasteurized	Northwest China (GS)	Summer	12.27
46	Y7-0824	Pasteurized	Northwest China (GS)	Summer	13.43
47	Y7-0825	Pasteurized	Northwest China (GS)	Summer	7.42
48	Y7-0827	Pasteurized	Northwest China (GS)	Summer	<5
49	Y7-0828	Pasteurized	Northwest China (GS)	Summer	6.41
50	Y7-0829	Pasteurized	Northwest China (GS)	Summer	9.24
51	Y7-0830	Pasteurized	Northwest China (GS)	Summer	6.65
52	Y7-0901	Pasteurized	Northwest China (GS)	Summer	<5
53	Y7-0902	Pasteurized	Northwest China (GS)	Summer	6.93
54	Y7-0903	Pasteurized	Northwest China (GS)	Summer	8.79
55	Y7-0904	Pasteurized	Northwest China (GS)	Summer	8.54
56	Y7-0905	Pasteurized	Northwest China (GS)	Summer	8.44
57	Y7-0906	Pasteurized	Northwest China (GS)	Summer	5.64
58	Y7-0908	Pasteurized	Northwest China (GS)	Summer	<5
59	Y7-0909	Pasteurized	Northwest China (GS)	Summer	<5
60	Y7-0910	Pasteurized	Northwest China (GS)	Summer	<5
61	Y7-0911	Pasteurized	Northwest China (GS)	Summer	<5
62	Y7-0912	Pasteurized	Northwest China (GS)	Summer	5.73
63	Y7-0913	Pasteurized	Northwest China (GS)	Summer	11.33
64	Y7-0914	Pasteurized	Northwest China (GS)	Summer	5.10

Serial Number	Sample ID	Milk type	Region (province) sampled	Season	AFM1 concentration (ng/L)
65	Y7-0915	Pasteurized	Northwest China (GS)	Summer	<5
66	Y7-0916	Pasteurized	Northwest China (GS)	Summer	8.89
67	Y7-0917	Pasteurized	Northwest China (GS)	Summer	7.00
68	Y7-0918	Pasteurized	Northwest China (GS)	Summer	5.04
69	Y7-0919	Pasteurized	Northwest China (GS)	Summer	<5
70	Y7-0920	Pasteurized	Northwest China (GS)	Summer	<5
71	Y7-0921	Pasteurized	Northwest China (GS)	Summer	<5
72	Y7-0922	Pasteurized	Northwest China (GS)	Summer	6.35
73	Y7-0924	Pasteurized	Northwest China (GS)	Summer	7.58
74	Y7-0925	Pasteurized	Northwest China (GS)	Summer	7.10
75	Y7-0926	Pasteurized	Northwest China (GS)	Summer	5.28
76	Y7-0927	Pasteurized	Northwest China (GS)	Summer	6.38
77	Y7-0928	Pasteurized	Northwest China (GS)	Summer	5.61
78	Y7-0929	Pasteurized	Northwest China (GS)	Summer	<5
79	B6-0828	Pasteurized	Northwest China (NX)	Summer	7.98
80	B6-0829	Pasteurized	Northwest China (NX)	Summer	6.07
81	B6-0830	Pasteurized	Northwest China (NX)	Summer	6.49
82	B6-0831	Pasteurized	Northwest China (NX)	Summer	5.49
83	B6-0901	Pasteurized	Northwest China (NX)	Summer	6.59
84	B6-0902	Pasteurized	Northwest China (NX)	Summer	5.02
85	B6-0903	Pasteurized	Northwest China (NX)	Summer	6.56
86	B6-0904	Pasteurized	Northwest China (NX)	Summer	8.64
87	B6-0905	Pasteurized	Northwest China (NX)	Summer	9.15
88	B6-0906	Pasteurized	Northwest China (NX)	Summer	8.03
89	B6-0908	Pasteurized	Northwest China (NX)	Summer	<5
90	B6-0909	Pasteurized	Northwest China (NX)	Summer	5.53
91	B6-0910	Pasteurized	Northwest China (NX)	Summer	<5
92	B6-0911	Pasteurized	Northwest China (NX)	Summer	<5
93	B6-0912	Pasteurized	Northwest China (NX)	Summer	5.22
94	B6-0913	Pasteurized	Northwest China (NX)	Summer	6.65
95	B6-0914	Pasteurized	Northwest China (NX)	Summer	7.60
96	B6-0915	Pasteurized	Northwest China (NX)	Summer	13.51
97	B6-0916	Pasteurized	Northwest China (NX)	Summer	11.14
98	B6-0917	Pasteurized	Northwest China (NX)	Summer	12.35
99	B6-0918	Pasteurized	Northwest China (NX)	Summer	13.33
100	B6-0919	Pasteurized	Northwest China (NX)	Summer	10.70
101	B6-0920	Pasteurized	Northwest China (NX)	Summer	8.89
102	B6-0921	Pasteurized	Northwest China (NX)	Summer	12.75
103	B6-0922	Pasteurized	Northwest China (NX)	Summer	12.10
104	B6-0923	Pasteurized	Northwest China (NX)	Summer	11.66

Serial Number	Sample ID	Milk type	Region (province) sampled	Season	AFM1 concentration (ng/L)
105	B6-0924	Pasteurized	Northwest China (NX)	Summer	7.28
106	B6-0925	Pasteurized	Northwest China (NX)	Summer	8.21
107	B6-0926	Pasteurized	Northwest China (NX)	Summer	7.96
108	X3-0901	Pasteurized	Northwest China (NX)	Summer	5.09
109	X3-0902	Pasteurized	Northwest China (NX)	Summer	5.70
110	X3-0903	Pasteurized	Northwest China (NX)	Summer	6.87
111	X3-0904	Pasteurized	Northwest China (NX)	Summer	5.40
112	X3-0905	Pasteurized	Northwest China (NX)	Summer	11.28
113	X3-0906	Pasteurized	Northwest China (NX)	Summer	7.58
114	X3-0907	Pasteurized	Northwest China (NX)	Summer	5.38
115	X3-0908	Pasteurized	Northwest China (NX)	Summer	<5
116	X3-0909	Pasteurized	Northwest China (NX)	Summer	<5
117	X3-0911	Pasteurized	Northwest China (NX)	Summer	6.99
118	X3-0912	Pasteurized	Northwest China (NX)	Summer	11.74
119	X3-0913	Pasteurized	Northwest China (NX)	Summer	16.89
120	X3-0914	Pasteurized	Northwest China (NX)	Summer	12.63
121	X3-0915	Pasteurized	Northwest China (NX)	Summer	8.37
122	X3-0917	Pasteurized	Northwest China (NX)	Summer	7.05
123	X3-0918	Pasteurized	Northwest China (NX)	Summer	21.46
124	X3-0919	Pasteurized	Northwest China (NX)	Summer	22.58
125	X3-0921	Pasteurized	Northwest China (NX)	Summer	11.94
126	X3-0923	Pasteurized	Northwest China (NX)	Summer	9.45
127	X3-0924	Pasteurized	Northwest China (NX)	Summer	7.17
128	X3-0925	Pasteurized	Northwest China (NX)	Summer	6.85
129	X3-0926	Pasteurized	Northwest China (NX)	Summer	5.72
130	X3-0927	Pasteurized	Northwest China (NX)	Summer	14.51
131	X3-0928	Pasteurized	Northwest China (NX)	Summer	12.66
132	X3-0929	Pasteurized	Northwest China (NX)	Summer	16.09
133	X3-0930	Pasteurized	Northwest China (NX)	Summer	16.71
134	C180-0801	UHT	Northwest China (GS)	Summer	7.74
135	C180-0804	UHT	Northwest China (GS)	Summer	<5
136	C180-0807	UHT	Northwest China (GS)	Summer	<5
137	C180-0818	UHT	Northwest China (GS)	Summer	<5
138	C180-0901	UHT	Northwest China (GS)	Summer	6.48
139	C180-0909	UHT	Northwest China (GS)	Summer	7.81
140	C180-0910	UHT	Northwest China (GS)	Summer	8.07
141	C180-0912	UHT	Northwest China (GS)	Summer	19.56
142	C180-0913	UHT	Northwest China (GS)	Summer	12.11
143	C180-0925	UHT	Northwest China (GS)	Summer	8.30
144	C180-0928	UHT	Northwest China (GS)	Summer	7.84

Serial Number	Sample ID	Milk type	Region (province) sampled	Season	AFM1 concentration (ng/L)
145	C180-0929	UHT	Northwest China (GS)	Summer	8.10
146	F180-0801	UHT	Northwest China (GS)	Summer	5.62
147	F180-0804	UHT	Northwest China (GS)	Summer	6.34
148	F180-0809	UHT	Northwest China (GS)	Summer	<5
149	F180-0812	UHT	Northwest China (GS)	Summer	6.95
150	F180-0822	UHT	Northwest China (GS)	Summer	5.57
151	F180-0916	UHT	Northwest China (GS)	Summer	5.16
152	F180-0918	UHT	Northwest China (GS)	Summer	6.80
153	F180-0921	UHT	Northwest China (GS)	Summer	5.46
154	J180-0806	UHT	Northwest China (GS)	Summer	<5
155	J180-0821	UHT	Northwest China (GS)	Summer	<5
156	J180-0823	UHT	Northwest China (GS)	Summer	<5
157	J180-0902	UHT	Northwest China (GS)	Summer	9.26
158	J180-0904	UHT	Northwest China (GS)	Summer	10.67
159	J180-0907	UHT	Northwest China (GS)	Summer	<5
160	J180-0912	UHT	Northwest China (GS)	Summer	<5
161	M180-0802	UHT	Northwest China (GS)	Summer	<5
162	M180-0805	UHT	Northwest China (GS)	Summer	<5
163	M180-0807	UHT	Northwest China (GS)	Summer	<5
164	M180-0822	UHT	Northwest China (GS)	Summer	<5
165	M180-0824	UHT	Northwest China (GS)	Summer	12.11
166	M180-0908	UHT	Northwest China (GS)	Summer	<5
167	M180-0909	UHT	Northwest China (GS)	Summer	5.53
168	M180-0914	UHT	Northwest China (GS)	Summer	5.93
169	M180-0921	UHT	Northwest China (GS)	Summer	7.43
170	M180-0925	UHT	Northwest China (GS)	Summer	5.87
171	M180-0926	UHT	Northwest China (GS)	Summer	<5
172	M180-0927	UHT	Northwest China (GS)	Summer	<5
173	A180-0805	UHT	Northwest China (NX)	Summer	<5
174	A180-0807	UHT	Northwest China (NX)	Summer	<5
175	A180-0816	UHT	Northwest China (NX)	Summer	9.07
176	A180-0818	UHT	Northwest China (NX)	Summer	<5
177	A180-0822	UHT	Northwest China (NX)	Summer	<5
178	A180-0825	UHT	Northwest China (NX)	Summer	<5
179	A180-0826	UHT	Northwest China (NX)	Summer	5.21
180	A180-0902	UHT	Northwest China (NX)	Summer	6.30
181	A180-0907	UHT	Northwest China (NX)	Summer	8.33
182	A180-0910	UHT	Northwest China (NX)	Summer	<5
183	A180-0920	UHT	Northwest China (NX)	Summer	<5
184	A180-0926	UHT	Northwest China (NX)	Summer	5.83

Serial Number	Sample ID	Milk type	Region (province) sampled	Season	AFM1 concentration (ng/L)
185	J180-0801	UHT	Northwest China (NX)	Summer	6.67
186	J180-0802	UHT	Northwest China (NX)	Summer	11.58
187	J180-0808	UHT	Northwest China (NX)	Summer	<5
188	J180-0815	UHT	Northwest China (NX)	Summer	<5
189	J180-0816	UHT	Northwest China (NX)	Summer	6.75
190	J180-0818	UHT	Northwest China (NX)	Summer	<5
191	J180-0902	UHT	Northwest China (NX)	Summer	5.49
192	J180-0903	UHT	Northwest China (NX)	Summer	9.89
193	J180-0908	UHT	Northwest China (NX)	Summer	<5
194	J180-0913	UHT	Northwest China (NX)	Summer	13.67
195	J180-0910	UHT	Northwest China (NX)	Summer	6.00
196	J180-0914	UHT	Northwest China (NX)	Summer	7.88
197	J180-0919	UHT	Northwest China (NX)	Summer	8.96
198	M180-0807	UHT	Northwest China (NX)	Summer	10.36
199	M180-0808	UHT	Northwest China (NX)	Summer	8.96
200	M180-0811	UHT	Northwest China (NX)	Summer	<5
201	M180-0816	UHT	Northwest China (NX)	Summer	<5
202	M180-0901	UHT	Northwest China (NX)	Summer	5.77
203	M180-0904	UHT	Northwest China (NX)	Summer	<5
204	M180-0906	UHT	Northwest China (NX)	Summer	6.59
205	M180-0909	UHT	Northwest China (NX)	Summer	<5
206	M180-0913	UHT	Northwest China (NX)	Summer	7.23
207	M180-0924	UHT	Northwest China (NX)	Summer	10.03
208	M180-0930	UHT	Northwest China (NX)	Summer	6.00
209	D5-1210	Pasteurized	Northwest China (GS)	Winter	10.58
210	D5-1212	Pasteurized	Northwest China (GS)	Winter	12.52
211	D5-1214	Pasteurized	Northwest China (GS)	Winter	12.06
212	D5-1216	Pasteurized	Northwest China (GS)	Winter	6.01
213	D5-1218	Pasteurized	Northwest China (GS)	Winter	17.64
214	D5-1220	Pasteurized	Northwest China (GS)	Winter	8.49
215	D5-1224	Pasteurized	Northwest China (GS)	Winter	12.43
216	D5-1226	Pasteurized	Northwest China (GS)	Winter	5.11
217	D5-1227	Pasteurized	Northwest China (GS)	Winter	<5
218	D5-1228	Pasteurized	Northwest China (GS)	Winter	<5
219	D5-1230	Pasteurized	Northwest China (GS)	Winter	<5
220	D5-0101	Pasteurized	Northwest China (GS)	Winter	8.46
221	D5-0103	Pasteurized	Northwest China (GS)	Winter	<5
222	D5-0105	Pasteurized	Northwest China (GS)	Winter	5.45
223	D5-0107	Pasteurized	Northwest China (GS)	Winter	<5
224	D5-0109	Pasteurized	Northwest China (GS)	Winter	<5

Serial Number	Sample ID	Milk type	Region (province) sampled	Season	AFM1 concentration (ng/L)
225	D5-0111	Pasteurized	Northwest China (GS)	Winter	<5
226	D5-0113	Pasteurized	Northwest China (GS)	Winter	<5
227	D5-0115	Pasteurized	Northwest China (GS)	Winter	5.49
228	D5-0116	Pasteurized	Northwest China (GS)	Winter	6.04
229	D5-0117	Pasteurized	Northwest China (GS)	Winter	<5
230	D5-0118	Pasteurized	Northwest China (GS)	Winter	5.18
231	D5-0120	Pasteurized	Northwest China (GS)	Winter	<5
232	D5-0121	Pasteurized	Northwest China (GS)	Winter	<5
233	D5-0127	Pasteurized	Northwest China (GS)	Winter	5.05
234	D5-0128	Pasteurized	Northwest China (GS)	Winter	<5
235	D5-0129	Pasteurized	Northwest China (GS)	Winter	5.45
236	D5-0130	Pasteurized	Northwest China (GS)	Winter	6.44
237	Y7-1202	Pasteurized	Northwest China (GS)	Winter	11.00
238	Y7-1204	Pasteurized	Northwest China (GS)	Winter	10.70
239	Y7-1206	Pasteurized	Northwest China (GS)	Winter	10.19
240	Y7-1208	Pasteurized	Northwest China (GS)	Winter	10.64
241	Y7-1210	Pasteurized	Northwest China (GS)	Winter	7.68
242	Y7-1212	Pasteurized	Northwest China (GS)	Winter	7.52
243	Y7-1214	Pasteurized	Northwest China (GS)	Winter	6.64
244	Y7-1216	Pasteurized	Northwest China (GS)	Winter	8.30
245	Y7-1218	Pasteurized	Northwest China (GS)	Winter	8.08
246	Y7-1220	Pasteurized	Northwest China (GS)	Winter	7.42
247	Y7-1222	Pasteurized	Northwest China (GS)	Winter	7.58
248	Y7-1224	Pasteurized	Northwest China (GS)	Winter	8.24
249	Y7-1226	Pasteurized	Northwest China (GS)	Winter	5.58
250	Y7-1227	Pasteurized	Northwest China (GS)	Winter	5.45
251	Y7-1228	Pasteurized	Northwest China (GS)	Winter	<5
252	Y7-1229	Pasteurized	Northwest China (GS)	Winter	5.83
253	Y7-1230	Pasteurized	Northwest China (GS)	Winter	<5
254	Y7-0101	Pasteurized	Northwest China (GS)	Winter	5.51
255	Y7-0103	Pasteurized	Northwest China (GS)	Winter	<5
256	Y7-0105	Pasteurized	Northwest China (GS)	Winter	5.73
257	Y7-0108	Pasteurized	Northwest China (GS)	Winter	6.40
258	Y7-0115	Pasteurized	Northwest China (GS)	Winter	9.15
259	Y7-0116	Pasteurized	Northwest China (GS)	Winter	11.65
260	Y7-0117	Pasteurized	Northwest China (GS)	Winter	17.27
261	Y7-0119	Pasteurized	Northwest China (GS)	Winter	9.13
262	Y7-0121	Pasteurized	Northwest China (GS)	Winter	7.91
263	Y7-0122	Pasteurized	Northwest China (GS)	Winter	5.64
264	Y7-0123	Pasteurized	Northwest China (GS)	Winter	5.64

Serial Number	Sample ID	Milk type	Region (province) sampled	Season	AFM1 concentration (ng/L)
265	Y7-0125	Pasteurized	Northwest China (GS)	Winter	9.59
266	Y7-0126	Pasteurized	Northwest China (GS)	Winter	8.93
267	Y7-0127	Pasteurized	Northwest China (GS)	Winter	5.09
268	Y7-0129	Pasteurized	Northwest China (GS)	Winter	9.22
269	Y7-0130	Pasteurized	Northwest China (GS)	Winter	8.58
270	B6-1201	Pasteurized	Northwest China (NX)	Winter	14.93
271	B6-1202	Pasteurized	Northwest China (NX)	Winter	18.38
272	B6-1203	Pasteurized	Northwest China (NX)	Winter	14.62
273	B6-1204	Pasteurized	Northwest China (NX)	Winter	18.84
274	B6-1205	Pasteurized	Northwest China (NX)	Winter	16.94
275	B6-1206	Pasteurized	Northwest China (NX)	Winter	18.11
276	B6-1207	Pasteurized	Northwest China (NX)	Winter	18.72
277	B6-1208	Pasteurized	Northwest China (NX)	Winter	16.90
278	B6-1209	Pasteurized	Northwest China (NX)	Winter	17.37
279	B6-1210	Pasteurized	Northwest China (NX)	Winter	12.80
280	B6-1211	Pasteurized	Northwest China (NX)	Winter	12.83
281	B6-1213	Pasteurized	Northwest China (NX)	Winter	12.95
282	B6-1215	Pasteurized	Northwest China (NX)	Winter	12.15
283	B6-1216	Pasteurized	Northwest China (NX)	Winter	13.85
284	B6-1217	Pasteurized	Northwest China (NX)	Winter	12.78
285	B6-1218	Pasteurized	Northwest China (NX)	Winter	12.27
286	B6-1221	Pasteurized	Northwest China (NX)	Winter	11.57
287	B6-1224	Pasteurized	Northwest China (NX)	Winter	10.99
288	B6-1226	Pasteurized	Northwest China (NX)	Winter	9.61
289	B6-1228	Pasteurized	Northwest China (NX)	Winter	11.11
290	B6-1230	Pasteurized	Northwest China (NX)	Winter	14.08
291	B6-0101	Pasteurized	Northwest China (NX)	Winter	14.67
292	B6-0103	Pasteurized	Northwest China (NX)	Winter	13.75
293	B6-0105	Pasteurized	Northwest China (NX)	Winter	10.69
294	B6-0107	Pasteurized	Northwest China (NX)	Winter	23.85
295	B6-0109	Pasteurized	Northwest China (NX)	Winter	12.31
296	B6-0111	Pasteurized	Northwest China (NX)	Winter	12.47
297	B6-0113	Pasteurized	Northwest China (NX)	Winter	11.67
298	B6-0115	Pasteurized	Northwest China (NX)	Winter	12.02
299	B6-0117	Pasteurized	Northwest China (NX)	Winter	12.41
300	B6-0118	Pasteurized	Northwest China (NX)	Winter	12.31
301	B6-0119	Pasteurized	Northwest China (NX)	Winter	9.77
302	B6-0121	Pasteurized	Northwest China (NX)	Winter	13.48
303	B6-0123	Pasteurized	Northwest China (NX)	Winter	11.41
304	B6-0125	Pasteurized	Northwest China (NX)	Winter	14.24

Serial Number	Sample ID	Milk type	Region (province) sampled	Season	AFM1 concentration (ng/L)
305	B6-0126	Pasteurized	Northwest China (NX)	Winter	12.60
306	B6-0127	Pasteurized	Northwest China (NX)	Winter	13.26
307	B6-0129	Pasteurized	Northwest China (NX)	Winter	13.67
308	B6-0131	Pasteurized	Northwest China (NX)	Winter	10.07
309	X3-1203	Pasteurized	Northwest China (NX)	Winter	10.07
310	X3-1208	Pasteurized	Northwest China (NX)	Winter	9.04
311	X3-1210	Pasteurized	Northwest China (NX)	Winter	8.00
312	X3-1212	Pasteurized	Northwest China (NX)	Winter	10.26
313	X3-1214	Pasteurized	Northwest China (NX)	Winter	11.15
314	X3-1215	Pasteurized	Northwest China (NX)	Winter	13.65
315	X3-1216	Pasteurized	Northwest China (NX)	Winter	12.76
316	X3-1218	Pasteurized	Northwest China (NX)	Winter	11.47
317	X3-1220	Pasteurized	Northwest China (NX)	Winter	12.58
318	X3-1222	Pasteurized	Northwest China (NX)	Winter	10.53
319	X3-1224	Pasteurized	Northwest China (NX)	Winter	6.68
320	X3-1226	Pasteurized	Northwest China (NX)	Winter	12.41
321	X3-1228	Pasteurized	Northwest China (NX)	Winter	12.17
322	X3-1230	Pasteurized	Northwest China (NX)	Winter	13.89
323	X3-1231	Pasteurized	Northwest China (NX)	Winter	12.97
324	X3-0101	Pasteurized	Northwest China (NX)	Winter	10.55
325	X3-0103	Pasteurized	Northwest China (NX)	Winter	12.85
326	X3-0105	Pasteurized	Northwest China (NX)	Winter	14.48
327	X3-0106	Pasteurized	Northwest China (NX)	Winter	16.84
328	X3-0108	Pasteurized	Northwest China (NX)	Winter	22.90
329	C180-1202	UHT	Northwest China (GS)	Winter	10.40
330	C180-1206	UHT	Northwest China (GS)	Winter	5.23
331	C180-1207	UHT	Northwest China (GS)	Winter	5.26
332	C180-1208	UHT	Northwest China (GS)	Winter	7.60
333	C180-1210	UHT	Northwest China (GS)	Winter	6.28
334	C180-1212	UHT	Northwest China (GS)	Winter	7.77
335	C180-1213	UHT	Northwest China (GS)	Winter	10.06
336	C180-1214	UHT	Northwest China (GS)	Winter	<5
337	C180-1223	UHT	Northwest China (GS)	Winter	<5
338	C180-1224	UHT	Northwest China (GS)	Winter	<5
339	C180-1221	UHT	Northwest China (GS)	Winter	6.28
340	C180-1227	UHT	Northwest China (GS)	Winter	<5
341	C180-1229	UHT	Northwest China (GS)	Winter	13.59
342	C180-1230	UHT	Northwest China (GS)	Winter	14.47
343	C180-0104	UHT	Northwest China (GS)	Winter	9.85
344	C180-0116	UHT	Northwest China (GS)	Winter	11.22

Serial Number	Sample ID	Milk type	Region (province) sampled	Season	AFM1 concentration (ng/L)
345	C180-0126	UHT	Northwest China (GS)	Winter	<5
346	F180-0101	UHT	Northwest China (GS)	Winter	6.97
347	F180-0104	UHT	Northwest China (GS)	Winter	7.52
348	F180-0105	UHT	Northwest China (GS)	Winter	6.02
349	F180-0106	UHT	Northwest China (GS)	Winter	8.31
350	F180-0107	UHT	Northwest China (GS)	Winter	8.92
351	F180-0129	UHT	Northwest China (GS)	Winter	<5
352	F180-1207	UHT	Northwest China (GS)	Winter	7.20
353	F180-1210	UHT	Northwest China (GS)	Winter	11.25
354	F180-1214	UHT	Northwest China (GS)	Winter	9.18
355	F180-1229	UHT	Northwest China (GS)	Winter	<5
356	J180-0107	UHT	Northwest China (GS)	Winter	10.38
357	J180-0109	UHT	Northwest China (GS)	Winter	6.60
358	J180-0120	UHT	Northwest China (GS)	Winter	8.31
359	J180-0121	UHT	Northwest China (GS)	Winter	<5
360	J180-0125	UHT	Northwest China (GS)	Winter	7.71
361	J180-1222	UHT	Northwest China (GS)	Winter	10.15
362	J180-1224	UHT	Northwest China (GS)	Winter	10.08
363	J180-1230	UHT	Northwest China (GS)	Winter	<5
364	M180-0103	UHT	Northwest China (GS)	Winter	6.26
365	M180-0107	UHT	Northwest China (GS)	Winter	16.19
366	M180-0109	UHT	Northwest China (GS)	Winter	13.68
367	M180-0130	UHT	Northwest China (GS)	Winter	14.10
368	M180-1207	UHT	Northwest China (GS)	Winter	11.11
369	M180-1209	UHT	Northwest China (GS)	Winter	9.57
370	M180-1210	UHT	Northwest China (GS)	Winter	8.36
371	M180-1224	UHT	Northwest China (GS)	Winter	5.37
372	A180-1205	UHT	Northwest China (NX)	Winter	<5
373	A180-1209	UHT	Northwest China (NX)	Winter	5.46
374	A180-1212	UHT	Northwest China (NX)	Winter	15.19
375	A180-1218	UHT	Northwest China (NX)	Winter	<5
376	A180-1225	UHT	Northwest China (NX)	Winter	<5
377	A180-1227	UHT	Northwest China (NX)	Winter	<5
378	A180-0101	UHT	Northwest China (NX)	Winter	<5
379	A180-0103	UHT	Northwest China (NX)	Winter	<5
380	A180-0105	UHT	Northwest China (NX)	Winter	<5
381	A180-0109	UHT	Northwest China (NX)	Winter	<5
382	A180-0111	UHT	Northwest China (NX)	Winter	15.24
383	A180-0115	UHT	Northwest China (NX)	Winter	<5
384	A180-1223	UHT	Northwest China (NX)	Winter	<5

Serial Number	Sample ID	Milk type	Region (province) sampled	Season	AFM1 concentration (ng/L)
385	A180-0124	UHT	Northwest China (NX)	Winter	5.13
386	A180-0130	UHT	Northwest China (NX)	Winter	<5
387	J180-1201	UHT	Northwest China (NX)	Winter	9.01
388	J180-1203	UHT	Northwest China (NX)	Winter	5.81
389	J180-1208	UHT	Northwest China (NX)	Winter	13.96
390	J180-1211	UHT	Northwest China (NX)	Winter	9.66
391	J180-1213	UHT	Northwest China (NX)	Winter	10.12
392	J180-1215	UHT	Northwest China (NX)	Winter	9.90
393	J180-1218	UHT	Northwest China (NX)	Winter	10.04
394	J180-1219	UHT	Northwest China (NX)	Winter	8.21
395	J180-1221	UHT	Northwest China (NX)	Winter	9.69
396	J180-1223	UHT	Northwest China (NX)	Winter	7.54
397	J180-0108	UHT	Northwest China (NX)	Winter	9.54
398	J180-0115	UHT	Northwest China (NX)	Winter	<5
399	J180-0123	UHT	Northwest China (NX)	Winter	<5
400	J180-0126	UHT	Northwest China (NX)	Winter	7.22
401	M180-1209	UHT	Northwest China (NX)	Winter	<5
402	M180-1223	UHT	Northwest China (NX)	Winter	<5
403	M180-1224	UHT	Northwest China (NX)	Winter	<5
404	M180-0101	UHT	Northwest China (NX)	Winter	11.39
405	M180-0103	UHT	Northwest China (NX)	Winter	6.00
406	M180-0104	UHT	Northwest China (NX)	Winter	7.59
407	M180-0105	UHT	Northwest China (NX)	Winter	7.57
408	M180-0107	UHT	Northwest China (NX)	Winter	6.11
409	M180-0130	UHT	Northwest China (NX)	Winter	16.01

Note: (1) Sample ID: the first character indicates the brand of the milk product, followed by numbers showing the story life (days). And then the last four numbers after "dash" are the sampling number. (2) UHT: ultra-high temperature. (3) In the sampling location column, GS is the abbreviation for Gansu province and NX for Ningxia Hui autonomous region, both located in northwestern China. (4) LOD = 5 ng/L.

Table S3. AFM1 concentration in two types of milk from south-central China.

Serial Number	Sample ID	Milk type	Region (province) sampled	Season	AFM1 concentration (ng/L)
1	L2-0820	Pasteurized	South-central China (HB)	Summer	31.64
2	L2-0821	Pasteurized	South-central China (HB)	Summer	48.18
3	L2-0822	Pasteurized	South-central China (HB)	Summer	22.93
4	L2-0823	Pasteurized	South-central China (HB)	Summer	17.53
5	L2-0824	Pasteurized	South-central China (HB)	Summer	15.89

Serial Number	Sample ID	Milk type	Region (province) sampled	Season	AFM1 concentration (ng/L)
6	L2-0825	Pasteurized	South-central China (HB)	Summer	21.07
7	L2-0826	Pasteurized	South-central China (HB)	Summer	20.14
8	L2-0827	Pasteurized	South-central China (HB)	Summer	33.02
9	L2-0828	Pasteurized	South-central China (HB)	Summer	15.55
10	L2-0829	Pasteurized	South-central China (HB)	Summer	22.63
11	L2-0830	Pasteurized	South-central China (HB)	Summer	15.53
12	L2-0831	Pasteurized	South-central China (HB)	Summer	15.53
13	L2-0901	Pasteurized	South-central China (HB)	Summer	25.86
14	L2-0902	Pasteurized	South-central China (HB)	Summer	29.92
15	L2-0903	Pasteurized	South-central China (HB)	Summer	37.69
16	L2-0904	Pasteurized	South-central China (HB)	Summer	38.99
17	L2-0905	Pasteurized	South-central China (HB)	Summer	23.89
18	L2-0906	Pasteurized	South-central China (HB)	Summer	52.08
19	L2-0907	Pasteurized	South-central China (HB)	Summer	31.75
20	L2-0908	Pasteurized	South-central China (HB)	Summer	30.02
21	L2-0909	Pasteurized	South-central China (HB)	Summer	28.76
22	L2-0910	Pasteurized	South-central China (HB)	Summer	48.48
23	L2-0911	Pasteurized	South-central China (HB)	Summer	31.35
24	L2-0912	Pasteurized	South-central China (HB)	Summer	27.04
25	L2-0913	Pasteurized	South-central China (HB)	Summer	20.67
26	L2-0914	Pasteurized	South-central China (HB)	Summer	25.42
27	L2-0915	Pasteurized	South-central China (HB)	Summer	19.16
28	L2-0916	Pasteurized	South-central China (HB)	Summer	23.11
29	L2-0917	Pasteurized	South-central China (HB)	Summer	25.56
30	L2-0918	Pasteurized	South-central China (HB)	Summer	22.20
31	L2-0919	Pasteurized	South-central China (HB)	Summer	25.64
32	L2-0920	Pasteurized	South-central China (HB)	Summer	23.07
33	L2-0921	Pasteurized	South-central China (HB)	Summer	16.74
34	L2-0922	Pasteurized	South-central China (HB)	Summer	24.39
35	L2-0923	Pasteurized	South-central China (HB)	Summer	16.07
36	L2-0924	Pasteurized	South-central China (HB)	Summer	22.52
37	L2-0925	Pasteurized	South-central China (HB)	Summer	23.68
38	L2-0926	Pasteurized	South-central China (HB)	Summer	23.23
39	L2-0927	Pasteurized	South-central China (HB)	Summer	23.07
40	L2-0928	Pasteurized	South-central China (HB)	Summer	17.58
41	L2-0929	Pasteurized	South-central China (HB)	Summer	20.71
42	L2-0930	Pasteurized	South-central China (HB)	Summer	24.35
43	S7-0820	Pasteurized	South-central China (HN)	Summer	<5
44	S7-0821	Pasteurized	South-central China (HN)	Summer	6.18
45	S7-0823	Pasteurized	South-central China (HN)	Summer	<5

Serial Number	Sample ID	Milk type	Region (province) sampled	Season	AFM1 concentration (ng/L)
46	S7-0824	Pasteurized	South-central China (HN)	Summer	9.57
47	S7-0825	Pasteurized	South-central China (HN)	Summer	11.66
48	S7-0826	Pasteurized	South-central China (HN)	Summer	9.35
49	S7-0828	Pasteurized	South-central China (HN)	Summer	5.93
50	S7-0829	Pasteurized	South-central China (HN)	Summer	8.06
51	S7-0830	Pasteurized	South-central China (HN)	Summer	9.23
52	S7-0831	Pasteurized	South-central China (HN)	Summer	10.47
53	S7-0901	Pasteurized	South-central China (HN)	Summer	<5
54	S7-0902	Pasteurized	South-central China (HN)	Summer	5.49
55	S7-0903	Pasteurized	South-central China (HN)	Summer	<5
56	S7-0904	Pasteurized	South-central China (HN)	Summer	8.90
57	S7-0905	Pasteurized	South-central China (HN)	Summer	<5
58	S7-0906	Pasteurized	South-central China (HN)	Summer	<5
59	S7-0907	Pasteurized	South-central China (HN)	Summer	<5
60	S7-0908	Pasteurized	South-central China (HN)	Summer	<5
61	S7-0909	Pasteurized	South-central China (HN)	Summer	<5
62	S7-0910	Pasteurized	South-central China (HN)	Summer	<5
63	S7-0911	Pasteurized	South-central China (HN)	Summer	<5
64	S7-0912	Pasteurized	South-central China (HN)	Summer	<5
65	S7-0913	Pasteurized	South-central China (HN)	Summer	8.53
66	S7-0914	Pasteurized	South-central China (HN)	Summer	<5
67	S7-0915	Pasteurized	South-central China (HN)	Summer	<5
68	S7-0916	Pasteurized	South-central China (HN)	Summer	<5
69	S7-0917	Pasteurized	South-central China (HN)	Summer	<5
70	S7-0918	Pasteurized	South-central China (HN)	Summer	<5
71	S7-0919	Pasteurized	South-central China (HN)	Summer	10.38
72	S7-0920	Pasteurized	South-central China (HN)	Summer	<5
73	S7-0921	Pasteurized	South-central China (HN)	Summer	6.30
74	S7-0922	Pasteurized	South-central China (HN)	Summer	<5
75	S7-0923	Pasteurized	South-central China (HN)	Summer	7.49
76	S7-0924	Pasteurized	South-central China (HN)	Summer	<5
77	S7-0925	Pasteurized	South-central China (HN)	Summer	6.41
78	S7-0926	Pasteurized	South-central China (HN)	Summer	<5
79	S7-0927	Pasteurized	South-central China (HN)	Summer	7.73
80	S7-0928	Pasteurized	South-central China (HN)	Summer	<5
81	S7-0929	Pasteurized	South-central China (HN)	Summer	5.72
82	S7-0930	Pasteurized	South-central China (HN)	Summer	9.09
83	S7-0803	Pasteurized	South-central China (HN)	Summer	6.88
84	S7-0804	Pasteurized	South-central China (HN)	Summer	9.22
85	S7-0805	Pasteurized	South-central China (HN)	Summer	14.04

Serial Number	Sample ID	Milk type	Region (province) sampled	Season	AFM1 concentration (ng/L)
86	S7-0807	Pasteurized	South-central China (HN)	Summer	13.68
87	S7-0808	Pasteurized	South-central China (HN)	Summer	7.28
88	S7-0809	Pasteurized	South-central China (HN)	Summer	14.18
89	S7-0810	Pasteurized	South-central China (HN)	Summer	8.13
90	S7-0813	Pasteurized	South-central China (HN)	Summer	9.85
91	S7-0814	Pasteurized	South-central China (HN)	Summer	7.26
92	Z5-0819	Pasteurized	South-central China (HN)	Summer	15.36
93	Z5-0820	Pasteurized	South-central China (HN)	Summer	16.57
94	Z5-0821	Pasteurized	South-central China (HN)	Summer	7.55
95	Z5-0822	Pasteurized	South-central China (HN)	Summer	6.81
96	Z5-0823	Pasteurized	South-central China (HN)	Summer	5.56
97	Z5-0824	Pasteurized	South-central China (HN)	Summer	7.21
98	Z5-0825	Pasteurized	South-central China (HN)	Summer	5.45
99	Z5-0826	Pasteurized	South-central China (HN)	Summer	5.88
100	Z5-0827	Pasteurized	South-central China (HN)	Summer	7.02
101	Z5-0828	Pasteurized	South-central China (HN)	Summer	<5
102	Z5-0829	Pasteurized	South-central China (HN)	Summer	<5
103	Z5-0830	Pasteurized	South-central China (HN)	Summer	5.21
104	Z5-0831	Pasteurized	South-central China (HN)	Summer	<5
105	Z5-0901	Pasteurized	South-central China (HN)	Summer	9.93
106	Z5-0902	Pasteurized	South-central China (HN)	Summer	<5
107	Z5-0903	Pasteurized	South-central China (HN)	Summer	<5
108	Z5-0904	Pasteurized	South-central China (HN)	Summer	5.45
109	Z5-0905	Pasteurized	South-central China (HN)	Summer	5
110	Z5-0906	Pasteurized	South-central China (HN)	Summer	<5
111	Z5-0907	Pasteurized	South-central China (HN)	Summer	<5
112	Z5-0908	Pasteurized	South-central China (HN)	Summer	5.13
113	Z5-0909	Pasteurized	South-central China (HN)	Summer	5.21
114	Z5-0910	Pasteurized	South-central China (HN)	Summer	<5
115	Z5-0911	Pasteurized	South-central China (HN)	Summer	<5
116	Z5-0912	Pasteurized	South-central China (HN)	Summer	<5
117	Z5-0913	Pasteurized	South-central China (HN)	Summer	14.4
118	Z5-0914	Pasteurized	South-central China (HN)	Summer	<5
119	Z5-0915	Pasteurized	South-central China (HN)	Summer	<5
120	Z5-0916	Pasteurized	South-central China (HN)	Summer	13.28
121	Z5-0917	Pasteurized	South-central China (HN)	Summer	15.44
122	Z5-0918	Pasteurized	South-central China (HN)	Summer	12.89
123	Z5-0919	Pasteurized	South-central China (HN)	Summer	13.30
124	Z5-0920	Pasteurized	South-central China (HN)	Summer	14.02
125	Z5-0921	Pasteurized	South-central China (HN)	Summer	13.25

Serial Number	Sample ID	Milk type	Region (province) sampled	Season	AFM1 concentration (ng/L)
126	Z5-0922	Pasteurized	South-central China (HN)	Summer	13.46
127	Z5-0923	Pasteurized	South-central China (HN)	Summer	21.96
128	Z5-0924	Pasteurized	South-central China (HN)	Summer	16.95
129	Z5-0925	Pasteurized	South-central China (HN)	Summer	17.95
130	Z5-0926	Pasteurized	South-central China (HN)	Summer	20.67
131	Z5-0927	Pasteurized	South-central China (HN)	Summer	18.45
132	Z5-0928	Pasteurized	South-central China (HN)	Summer	8.21
133	Z5-0929	Pasteurized	South-central China (HN)	Summer	14.34
134	A180-0801	UHT	South-central China (HB)	Summer	<5
135	A180-0811	UHT	South-central China (HB)	Summer	9.42
136	A180-0814	UHT	South-central China (HB)	Summer	9.76
137	A180-0818	UHT	South-central China (HB)	Summer	6.98
138	A180-0819	UHT	South-central China (HB)	Summer	9.64
139	A180-0820	UHT	South-central China (HB)	Summer	8.10
140	A180-0823	UHT	South-central China (HB)	Summer	8.53
141	A180-0824	UHT	South-central China (HB)	Summer	10.30
142	A180-0828	UHT	South-central China (HB)	Summer	<5
143	A180-0829	UHT	South-central China (HB)	Summer	6.82
144	A180-0830	UHT	South-central China (HB)	Summer	9.64
145	A180-0831	UHT	South-central China (HB)	Summer	7.99
146	A180-0905	UHT	South-central China (HB)	Summer	11.66
147	A180-0907	UHT	South-central China (HB)	Summer	7.28
148	A180-0909	UHT	South-central China (HB)	Summer	5.52
149	A180-0911	UHT	South-central China (HB)	Summer	8.87
150	A180-0913	UHT	South-central China (HB)	Summer	<5
151	A180-0914	UHT	South-central China (HB)	Summer	7.80
152	A180-0917	UHT	South-central China (HB)	Summer	8.94
153	A180-0920	UHT	South-central China (HB)	Summer	8.91
154	A180-0921	UHT	South-central China (HB)	Summer	9.35
155	A180-0923	UHT	South-central China (HB)	Summer	6.65
156	A180-0925	UHT	South-central China (HB)	Summer	13.44
157	A180-0927	UHT	South-central China (HB)	Summer	<5
158	G180-0801	UHT	South-central China (HB)	Summer	9.07
159	G180-0802	UHT	South-central China (HB)	Summer	<5
160	G180-0804	UHT	South-central China (HB)	Summer	5.33
161	G180-0809	UHT	South-central China (HB)	Summer	5.91
162	G180-0816	UHT	South-central China (HB)	Summer	<5
163	G180-0904	UHT	South-central China (HB)	Summer	<5
164	G180-0928	UHT	South-central China (HB)	Summer	<5
165	G180-0929	UHT	South-central China (HB)	Summer	<5

Serial Number	Sample ID	Milk type	Region (province) sampled	Season	AFM1 concentration (ng/L)
166	J180-0801	UHT	South-central China (HB)	Summer	6.19
167	J180-0808	UHT	South-central China (HB)	Summer	7.03
168	J180-0811	UHT	South-central China (HB)	Summer	5.07
169	J180-0813	UHT	South-central China (HB)	Summer	<5
170	J180-0819	UHT	South-central China (HB)	Summer	<5
171	J180-0831	UHT	South-central China (HB)	Summer	<5
172	J180-0901	UHT	South-central China (HB)	Summer	7.44
173	J180-0910	UHT	South-central China (HB)	Summer	<5
174	J180-0913	UHT	South-central China (HB)	Summer	9.42
175	J180-0918	UHT	South-central China (HB)	Summer	<5
176	J180-0927	UHT	South-central China (HB)	Summer	12.94
177	M180-0801	UHT	South-central China (HB)	Summer	11.57
178	M180-0803	UHT	South-central China (HB)	Summer	<5
179	M180-0804	UHT	South-central China (HB)	Summer	5.71
180	M180-0809	UHT	South-central China (HB)	Summer	<5
181	M180-0812	UHT	South-central China (HB)	Summer	13.00
182	M180-0813	UHT	South-central China (HB)	Summer	<5
183	M180-0817	UHT	South-central China (HB)	Summer	8.57
184	M180-0820	UHT	South-central China (HB)	Summer	<5
185	M180-0821	UHT	South-central China (HB)	Summer	<5
186	M180-0825	UHT	South-central China (HB)	Summer	6.55
187	M180-0826	UHT	South-central China (HB)	Summer	5.71
188	M180-0827	UHT	South-central China (HB)	Summer	16.17
189	M180-0828	UHT	South-central China (HB)	Summer	9.04
190	M180-0829	UHT	South-central China (HB)	Summer	6.77
191	M180-0830	UHT	South-central China (HB)	Summer	<5
192	M180-0831	UHT	South-central China (HB)	Summer	11.21
193	M180-0901	UHT	South-central China (HB)	Summer	<5
194	M180-0902	UHT	South-central China (HB)	Summer	5.34
195	M180-0903	UHT	South-central China (HB)	Summer	11.30
196	M180-0904	UHT	South-central China (HB)	Summer	7.80
197	M180-0910	UHT	South-central China (HB)	Summer	<5
198	M180-0912	UHT	South-central China (HB)	Summer	9.04
199	M180-0913	UHT	South-central China (HB)	Summer	5.71
200	M180-0915	UHT	South-central China (HB)	Summer	<5
201	M180-0916	UHT	South-central China (HB)	Summer	<5
202	M180-0928	UHT	South-central China (HB)	Summer	7.57
203	A180-0803	UHT	South-central China (HN)	Summer	<5
204	A180-0804	UHT	South-central China (HN)	Summer	<5
205	A180-0805	UHT	South-central China (HN)	Summer	<5

Serial Number	Sample ID	Milk type	Region (province) sampled	Season	AFM1 concentration (ng/L)
206	A180-0808	UHT	South-central China (HN)	Summer	8.50
207	A180-0809	UHT	South-central China (HN)	Summer	7.72
208	A180-0811	UHT	South-central China (HN)	Summer	<5
209	A180-0817	UHT	South-central China (HN)	Summer	7.34
210	A180-0818	UHT	South-central China (HN)	Summer	6.84
211	A180-0825	UHT	South-central China (HN)	Summer	10.85
212	A180-0826	UHT	South-central China (HN)	Summer	6.19
213	A180-0828	UHT	South-central China (HN)	Summer	11.57
214	A180-0904	UHT	South-central China (HN)	Summer	9.78
215	A180-0905	UHT	South-central China (HN)	Summer	6.51
216	A180-0906	UHT	South-central China (HN)	Summer	7.76
217	A180-0908	UHT	South-central China (HN)	Summer	7.79
218	A180-0913	UHT	South-central China (HN)	Summer	<5
219	A180-0916	UHT	South-central China (HN)	Summer	8.90
220	A180-0918	UHT	South-central China (HN)	Summer	6.02
221	A180-0919	UHT	South-central China (HN)	Summer	5.88
222	A180-0920	UHT	South-central China (HN)	Summer	5.01
223	A180-0921	UHT	South-central China (HN)	Summer	5.44
224	A180-0922	UHT	South-central China (HN)	Summer	7.33
225	A180-0923	UHT	South-central China (HN)	Summer	9.76
226	A180-0925	UHT	South-central China (HN)	Summer	6.56
227	A180-0926	UHT	South-central China (HN)	Summer	9.60
228	A180-0927	UHT	South-central China (HN)	Summer	6.65
229	A180-0928	UHT	South-central China (HN)	Summer	8.48
230	A180-0929	UHT	South-central China (HN)	Summer	5.76
231	A180-0930	UHT	South-central China (HN)	Summer	5.93
232	J180-0803	UHT	South-central China (HN)	Summer	5.79
233	J180-0808	UHT	South-central China (HN)	Summer	6.35
234	J180-0809	UHT	South-central China (HN)	Summer	<5
235	J180-0820	UHT	South-central China (HN)	Summer	5.13
236	J180-0823	UHT	South-central China (HN)	Summer	6.94
237	J180-0824	UHT	South-central China (HN)	Summer	6.77
238	J180-0825	UHT	South-central China (HN)	Summer	7.8
239	J180-0826	UHT	South-central China (HN)	Summer	<5
240	J180-0901	UHT	South-central China (HN)	Summer	8.82
241	J180-0909	UHT	South-central China (HN)	Summer	5.97
242	J180-0910	UHT	South-central China (HN)	Summer	5.28
243	J180-0912	UHT	South-central China (HN)	Summer	5.95
244	J180-0913	UHT	South-central China (HN)	Summer	9.34
245	J180-0914	UHT	South-central China (HN)	Summer	8.28

Serial Number	Sample ID	Milk type	Region (province) sampled	Season	AFM1 concentration (ng/L)
246	J180-0915	UHT	South-central China (HN)	Summer	5.34
247	J180-0920	UHT	South-central China (HN)	Summer	5.71
248	J180-0921	UHT	South-central China (HN)	Summer	7.18
249	J180-0922	UHT	South-central China (HN)	Summer	8.74
250	J180-0927	UHT	South-central China (HN)	Summer	6.07
251	J180-0928	UHT	South-central China (HN)	Summer	9.52
252	M180-0816	UHT	South-central China (HN)	Summer	9.21
253	M180-0819	UHT	South-central China (HN)	Summer	11.8
254	M180-0820	UHT	South-central China (HN)	Summer	7.29
255	M180-0905	UHT	South-central China (HN)	Summer	10.1
256	M180-0922	UHT	South-central China (HN)	Summer	9.84
257	M180-0927	UHT	South-central China (HN)	Summer	6.14
258	M180-0929	UHT	South-central China (HN)	Summer	<5
259	M180-0930	UHT	South-central China (HN)	Summer	<5
260	L2-1203	Pasteurized	South-central China (HB)	Winter	69.22
261	L2-1204	Pasteurized	South-central China (HB)	Winter	53.13
262	L2-1205	Pasteurized	South-central China (HB)	Winter	69.13
263	L2-1206	Pasteurized	South-central China (HB)	Winter	44.05
264	L2-1207	Pasteurized	South-central China (HB)	Winter	55.47
265	L2-1208	Pasteurized	South-central China (HB)	Winter	58.68
266	L2-1209	Pasteurized	South-central China (HB)	Winter	68.03
267	L2-1210	Pasteurized	South-central China (HB)	Winter	65.48
268	L2-1211	Pasteurized	South-central China (HB)	Winter	62.02
269	L2-1212	Pasteurized	South-central China (HB)	Winter	62.67
270	L2-1213	Pasteurized	South-central China (HB)	Winter	65.68
271	L2-1214	Pasteurized	South-central China (HB)	Winter	54.42
272	L2-1216	Pasteurized	South-central China (HB)	Winter	24.74
273	L2-1217	Pasteurized	South-central China (HB)	Winter	50.36
274	L2-1218	Pasteurized	South-central China (HB)	Winter	37.93
275	L2-1219	Pasteurized	South-central China (HB)	Winter	42.38
276	L2-1220	Pasteurized	South-central China (HB)	Winter	39.25
277	L2-1221	Pasteurized	South-central China (HB)	Winter	37.99
278	L2-1222	Pasteurized	South-central China (HB)	Winter	19.20
279	L2-1224	Pasteurized	South-central China (HB)	Winter	36.10
280	L2-1225	Pasteurized	South-central China (HB)	Winter	11.54
281	L2-1226	Pasteurized	South-central China (HB)	Winter	22.55
282	L2-1227	Pasteurized	South-central China (HB)	Winter	14.48
283	L2-1228	Pasteurized	South-central China (HB)	Winter	28.15
284	L2-1229	Pasteurized	South-central China (HB)	Winter	22.51
285	L2-1230	Pasteurized	South-central China (HB)	Winter	32.77

Serial Number	Sample ID	Milk type	Region (province) sampled	Season	AFM1 concentration (ng/L)
286	L2-0101	Pasteurized	South-central China (HB)	Winter	34.01
287	L2-0102	Pasteurized	South-central China (HB)	Winter	20.80
288	L2-0103	Pasteurized	South-central China (HB)	Winter	34.94
289	L2-0104	Pasteurized	South-central China (HB)	Winter	26.37
290	L2-0105	Pasteurized	South-central China (HB)	Winter	22.55
291	L2-0106	Pasteurized	South-central China (HB)	Winter	29.33
292	L2-0107	Pasteurized	South-central China (HB)	Winter	20.69
293	L2-0108	Pasteurized	South-central China (HB)	Winter	28.48
294	L2-0109	Pasteurized	South-central China (HB)	Winter	18.96
295	L2-0110	Pasteurized	South-central China (HB)	Winter	20.07
296	L2-0111	Pasteurized	South-central China (HB)	Winter	34.89
297	L2-0112	Pasteurized	South-central China (HB)	Winter	14.67
298	L2-0113	Pasteurized	South-central China (HB)	Winter	44.94
299	L2-0114	Pasteurized	South-central China (HB)	Winter	19.69
300	L2-0115	Pasteurized	South-central China (HB)	Winter	19.51
301	L2-0116	Pasteurized	South-central China (HB)	Winter	23.91
302	L2-0117	Pasteurized	South-central China (HB)	Winter	22.76
303	L2-0119	Pasteurized	South-central China (HB)	Winter	38.91
304	L2-0120	Pasteurized	South-central China (HB)	Winter	19.24
305	L2-0121	Pasteurized	South-central China (HB)	Winter	35.57
306	L2-0122	Pasteurized	South-central China (HB)	Winter	26.43
307	L2-0123	Pasteurized	South-central China (HB)	Winter	32.09
308	L2-0124	Pasteurized	South-central China (HB)	Winter	28.75
309	L2-0125	Pasteurized	South-central China (HB)	Winter	29.92
310	L2-0126	Pasteurized	South-central China (HB)	Winter	40.83
311	L2-0127	Pasteurized	South-central China (HB)	Winter	25.96
312	L2-0128	Pasteurized	South-central China (HB)	Winter	37.66
313	L2-0129	Pasteurized	South-central China (HB)	Winter	23.43
314	L2-0130	Pasteurized	South-central China (HB)	Winter	42.46
315	L2-0131	Pasteurized	South-central China (HB)	Winter	75.57
316	S7-1201	Pasteurized	South-central China (HN)	Winter	8.79
317	S7-1203	Pasteurized	South-central China (HN)	Winter	11.12
318	S7-1205	Pasteurized	South-central China (HN)	Winter	7.03
319	S7-1207	Pasteurized	South-central China (HN)	Winter	10.02
320	S7-1210	Pasteurized	South-central China (HN)	Winter	9.33
321	S7-1211	Pasteurized	South-central China (HN)	Winter	10.27
322	S7-1215	Pasteurized	South-central China (HN)	Winter	6.54
323	S7-1217	Pasteurized	South-central China (HN)	Winter	10.02
324	S7-1219	Pasteurized	South-central China (HN)	Winter	11.23
325	S7-1221	Pasteurized	South-central China (HN)	Winter	17.37

Serial Number	Sample ID	Milk type	Region (province) sampled	Season	AFM1 concentration (ng/L)
326	S7-1223	Pasteurized	South-central China (HN)	Winter	14.41
327	S7-1225	Pasteurized	South-central China (HN)	Winter	15.08
328	S7-1227	Pasteurized	South-central China (HN)	Winter	11.77
329	S7-1229	Pasteurized	South-central China (HN)	Winter	10.51
330	S7-1231	Pasteurized	South-central China (HN)	Winter	5.96
331	S7-0101	Pasteurized	South-central China (HN)	Winter	<5
332	S7-0103	Pasteurized	South-central China (HN)	Winter	5.46
333	S7-0105	Pasteurized	South-central China (HN)	Winter	<5
334	S7-0107	Pasteurized	South-central China (HN)	Winter	7.19
335	S7-0109	Pasteurized	South-central China (HN)	Winter	5.75
336	S7-0111	Pasteurized	South-central China (HN)	Winter	7.37
337	S7-0113	Pasteurized	South-central China (HN)	Winter	<5
338	S7-0115	Pasteurized	South-central China (HN)	Winter	6.85
339	S7-0117	Pasteurized	South-central China (HN)	Winter	6.64
340	S7-0119	Pasteurized	South-central China (HN)	Winter	11.36
341	Z5-1201	Pasteurized	South-central China (HN)	Winter	5.70
342	Z5-1203	Pasteurized	South-central China (HN)	Winter	13.54
343	Z5-1204	Pasteurized	South-central China (HN)	Winter	5.67
344	Z5-1206	Pasteurized	South-central China (HN)	Winter	15.30
345	Z5-1208	Pasteurized	South-central China (HN)	Winter	12.99
346	Z5-1210	Pasteurized	South-central China (HN)	Winter	6.87
347	Z5-1212	Pasteurized	South-central China (HN)	Winter	7.19
348	Z5-1214	Pasteurized	South-central China (HN)	Winter	10.02
349	Z5-1216	Pasteurized	South-central China (HN)	Winter	6.72
350	Z5-1218	Pasteurized	South-central China (HN)	Winter	6.81
351	Z5-1220	Pasteurized	South-central China (HN)	Winter	8.00
352	Z5-1222	Pasteurized	South-central China (HN)	Winter	9.24
353	Z5-1224	Pasteurized	South-central China (HN)	Winter	<5
354	Z5-1226	Pasteurized	South-central China (HN)	Winter	7.41
355	Z5-1228	Pasteurized	South-central China (HN)	Winter	6.70
356	Z5-1230	Pasteurized	South-central China (HN)	Winter	9.03
357	Z5-0101	Pasteurized	South-central China (HN)	Winter	8.46
358	Z5-0103	Pasteurized	South-central China (HN)	Winter	6.83
359	Z5-0105	Pasteurized	South-central China (HN)	Winter	7.29
360	Z5-0107	Pasteurized	South-central China (HN)	Winter	8.98
361	Z5-0109	Pasteurized	South-central China (HN)	Winter	7.53
362	Z5-0111	Pasteurized	South-central China (HN)	Winter	5.33
363	Z5-0113	Pasteurized	South-central China (HN)	Winter	<5
364	Z5-0115	Pasteurized	South-central China (HN)	Winter	9.26
365	Z5-0117	Pasteurized	South-central China (HN)	Winter	8.94

Serial Number	Sample ID	Milk type	Region (province) sampled	Season	AFM1 concentration (ng/L)
366	Z5-0119	Pasteurized	South-central China (HN)	Winter	10.33
367	A180-0104	UHT	South-central China (HB)	Winter	<5
368	A180-0106	UHT	South-central China (HB)	Winter	<5
369	A180-0111	UHT	South-central China (HB)	Winter	7.1
370	A180-0114	UHT	South-central China (HB)	Winter	<5
371	A180-0120	UHT	South-central China (HB)	Winter	<5
372	A180-0121	UHT	South-central China (HB)	Winter	5.89
373	A180-0125	UHT	South-central China (HB)	Winter	7.82
374	A180-0131	UHT	South-central China (HB)	Winter	5.34
375	A180-1201	UHT	South-central China (HB)	Winter	<5
376	A180-1204	UHT	South-central China (HB)	Winter	<5
377	A180-1206	UHT	South-central China (HB)	Winter	8.08
378	A180-1210	UHT	South-central China (HB)	Winter	7.07
379	A180-1213	UHT	South-central China (HB)	Winter	<5
380	A180-1216	UHT	South-central China (HB)	Winter	<5
381	A180-1217	UHT	South-central China (HB)	Winter	<5
382	A180-1224	UHT	South-central China (HB)	Winter	5.78
383	A180-1226	UHT	South-central China (HB)	Winter	<5
384	A180-1229	UHT	South-central China (HB)	Winter	<5
385	A180-1230	UHT	South-central China (HB)	Winter	19.01
386	G180-0105	UHT	South-central China (HB)	Winter	9.61
387	G180-0115	UHT	South-central China (HB)	Winter	7.71
388	G180-0116	UHT	South-central China (HB)	Winter	9.42
389	G180-0117	UHT	South-central China (HB)	Winter	10.40
390	G180-0121	UHT	South-central China (HB)	Winter	10.54
391	G180-1201	UHT	South-central China (HB)	Winter	<5
392	G180-1203	UHT	South-central China (HB)	Winter	6.14
393	G180-1207	UHT	South-central China (HB)	Winter	<5
394	G180-1216	UHT	South-central China (HB)	Winter	5.75
395	G180-1221	UHT	South-central China (HB)	Winter	<5
396	G180-1223	UHT	South-central China (HB)	Winter	<5
397	G180-1226	UHT	South-central China (HB)	Winter	9.80
398	G180-1227	UHT	South-central China (HB)	Winter	7.47
399	J180-0103	UHT	South-central China (HB)	Winter	6.18
400	J180-0108	UHT	South-central China (HB)	Winter	<5
401	J180-0109	UHT	South-central China (HB)	Winter	6.55
402	J180-0110	UHT	South-central China (HB)	Winter	<5
403	J180-0113	UHT	South-central China (HB)	Winter	<5
404	J180-0117	UHT	South-central China (HB)	Winter	8.96
405	J180-0119	UHT	South-central China (HB)	Winter	6.70

Serial Number	Sample ID	Milk type	Region (province) sampled	Season	AFM1 concentration (ng/L)
406	J180-0120	UHT	South-central China (HB)	Winter	<5
407	J180-0130	UHT	South-central China (HB)	Winter	6.75
408	J180-1202	UHT	South-central China (HB)	Winter	8.89
409	J180-1206	UHT	South-central China (HB)	Winter	<5
410	J180-1211	UHT	South-central China (HB)	Winter	5.43
411	J180-1213	UHT	South-central China (HB)	Winter	<5
412	J180-1215	UHT	South-central China (HB)	Winter	9.14
413	J180-1220	UHT	South-central China (HB)	Winter	<5
414	J180-1225	UHT	South-central China (HB)	Winter	7.21
415	J180-1226	UHT	South-central China (HB)	Winter	<5
416	J180-1229	UHT	South-central China (HB)	Winter	5.14
417	J180-1230	UHT	South-central China (HB)	Winter	5.51
418	M180-0101	UHT	South-central China (HB)	Winter	<5
419	M180-0103	UHT	South-central China (HB)	Winter	8.90
420	M180-0104	UHT	South-central China (HB)	Winter	7.03
421	M180-0106	UHT	South-central China (HB)	Winter	11.77
422	M180-0109	UHT	South-central China (HB)	Winter	<5
423	M180-0110	UHT	South-central China (HB)	Winter	18.70
424	M180-0112	UHT	South-central China (HB)	Winter	14.59
425	M180-0113	UHT	South-central China (HB)	Winter	8.60
426	M180-0116	UHT	South-central China (HB)	Winter	6.98
427	M180-0117	UHT	South-central China (HB)	Winter	12.94
428	M180-0118	UHT	South-central China (HB)	Winter	9.40
429	M180-0120	UHT	South-central China (HB)	Winter	<5
430	M180-0123	UHT	South-central China (HB)	Winter	10.71
431	M180-0125	UHT	South-central China (HB)	Winter	6.80
432	M180-0127	UHT	South-central China (HB)	Winter	12.22
433	M180-0128	UHT	South-central China (HB)	Winter	6.04
434	M180-0131	UHT	South-central China (HB)	Winter	8.53
435	M180-1202	UHT	South-central China (HB)	Winter	6.17
436	M180-1203	UHT	South-central China (HB)	Winter	5.02
437	M180-1204	UHT	South-central China (HB)	Winter	<5
438	M180-1205	UHT	South-central China (HB)	Winter	7.61
439	M180-1207	UHT	South-central China (HB)	Winter	6.50
440	M180-1211	UHT	South-central China (HB)	Winter	<5
441	M180-1212	UHT	South-central China (HB)	Winter	<5
442	M180-1214	UHT	South-central China (HB)	Winter	<5
443	M180-1215	UHT	South-central China (HB)	Winter	7.89
444	M180-1216	UHT	South-central China (HB)	Winter	<5
445	M180-1218	UHT	South-central China (HB)	Winter	5.35

Serial Number	Sample ID	Milk type	Region (province) sampled	Season	AFM1 concentration (ng/L)
446	M180-1219	UHT	South-central China (HB)	Winter	<5
447	M180-1222	UHT	South-central China (HB)	Winter	<5
448	M180-1225	UHT	South-central China (HB)	Winter	6.35
449	M180-1229	UHT	South-central China (HB)	Winter	<5
450	A180-0102	UHT	South-central China (HN)	Winter	<5
451	A180-0103	UHT	South-central China (HN)	Winter	<5
452	A180-0107	UHT	South-central China (HN)	Winter	<5
453	A180-0111	UHT	South-central China (HN)	Winter	<5
454	A180-0117	UHT	South-central China (HN)	Winter	7.32
455	A180-0126	UHT	South-central China (HN)	Winter	<5
456	A180-1203	UHT	South-central China (HN)	Winter	<5
457	A180-1207	UHT	South-central China (HN)	Winter	6.02
458	A180-1208	UHT	South-central China (HN)	Winter	5.99
459	A180-1209	UHT	South-central China (HN)	Winter	<5
460	A180-1211	UHT	South-central China (HN)	Winter	11.02
461	A180-1214	UHT	South-central China (HN)	Winter	<5
462	A180-1215	UHT	South-central China (HN)	Winter	7.52
463	A180-1217	UHT	South-central China (HN)	Winter	5.08
464	A180-1218	UHT	South-central China (HN)	Winter	13.07
465	A180-1220	UHT	South-central China (HN)	Winter	<5
466	A180-1221	UHT	South-central China (HN)	Winter	<5
467	A180-1227	UHT	South-central China (HN)	Winter	<5
468	A180-1228	UHT	South-central China (HN)	Winter	<5
469	J180-0111	UHT	South-central China (HN)	Winter	<5
470	J180-0112	UHT	South-central China (HN)	Winter	<5
471	J180-0117	UHT	South-central China (HN)	Winter	9.56
472	J180-0118	UHT	South-central China (HN)	Winter	<5
473	J180-0124	UHT	South-central China (HN)	Winter	6.82
474	J180-0129	UHT	South-central China (HN)	Winter	6.55
475	J180-1201	UHT	South-central China (HN)	Winter	6.89
476	J180-1204	UHT	South-central China (HN)	Winter	<5
477	J180-1205	UHT	South-central China (HN)	Winter	<5
478	J180-1212	UHT	South-central China (HN)	Winter	6.44
479	J180-1222	UHT	South-central China (HN)	Winter	<5
480	J180-1228	UHT	South-central China (HN)	Winter	7.71
481	M180-0102	UHT	South-central China (HN)	Winter	<5
482	M180-0105	UHT	South-central China (HN)	Winter	9.40
483	M180-0108	UHT	South-central China (HN)	Winter	5.46
484	M180-0111	UHT	South-central China (HN)	Winter	<5
485	M180-0114	UHT	South-central China (HN)	Winter	11.84

Serial Number	Sample ID	Milk type	Region (province) sampled	Season	AFM1 concentration (ng/L)
486	M180-0115	UHT	South-central China (HN)	Winter	9.89
487	M180-0122	UHT	South-central China (HN)	Winter	7.89
488	M180-0124	UHT	South-central China (HN)	Winter	9.49
489	M180-1201	UHT	South-central China (HN)	Winter	8.99
490	M180-1208	UHT	South-central China (HN)	Winter	<5
491	M180-1213	UHT	South-central China (HN)	Winter	<5
492	M180-1226	UHT	South-central China (HN)	Winter	<5
493	M180-1228	UHT	South-central China (HN)	Winter	<5

Note: (1) Sample ID: the first character indicates the brand of the milk product, followed by numbers showing the story life (days). And then the last four numbers after "dash" are the sampling number. (2) UHT: ultra-high temperature. (3) In the sampling location column, HB stands for Hubei province and HN for Hunan province, which are situated in south-central China. (4) LOD = 5 ng/L.

Table S4. Temperature and humidity in the two regions during the summer and winter surveys.

Province	Month	Temperature (°C)	Rainfall (mm/month)	Relative Humidity (%)
Northwest China in summer				
Gansu	Aug., 2023	21.7	29.7	52.8
	Sep., 2023	17.0	51.7	59.2
Ningxia	Aug., 2023	23.5	38.5	59.2
	Sep., 2023	18.6	21.7	64.6
Average		20.2	35.4	59.0
Central China in summer				
Hubei	Aug., 2023	28.1	140.8	75.4
	Sep., 2023	24.1	160.6	80.0
Hunan	Aug., 2023	28.6	128.5	76.9
	Sep., 2023	25.2	75.0	80.4
Average		26.5	126.2	78.2
Northwest China in winter				
Gansu	Dec., 2023	-3.7	1.8	45.8
	Jan., 2024	-4.8	0.6	44.4
Ningxia	Dec., 2023	-4.7	4.2	52.5
	Jan. 2024	-5.4	1.8	52.4
Average		-4.7	2.1	48.8
Central China in winter				
Hubei	Dec., 2023	6.2	46.2	74.8
	Jan., 2024	5.6	55.8	76.7
Hunan	Dec., 2023	8.1	22.8	71.9
	Jan., 2024	7.1	117.1	82.3
Average		6.8	60.5	76.4

Note: Climate data source: https://data.cma.cn/ (accessed on 5 January 2026).
